# Supplementary material for: Dual function of a highly conserved bacteriophage tail completion protein essential for bacteriophage infectivity
Source: Commun Biol. 2024 May 16;7:590. doi: 10.1038/s42003-024-06221-6 (PMC11099176; doi:10.1038/s42003-024-06221-6)
Supplement: Supplementary file 2 — Supplementary Information [file 42003_2024_6221_MOESM2_ESM.pdf]

Supplementary information

Dual function of a highly conserved bacteriophage tail completion protein essential for bacteriophage infectivity

Isabelle Auzat[1*], Malika Ouldali[2], Eric Jacquet[3], Beatrix Fauler[4], Thorsten Mielke[4] & Paulo Tavares[1]

[1]Université Paris-Saclay, CEA, CNRS, Institute for Integrative Biology of the Cell (I2BC), 91198 Gif-sur-Yvette, France

[2]Université Paris-Saclay, CEA, CNRS, Cryo-Electron Microscopy Facility, Institute for Integrative Biology of the Cell (I2BC), 91198 Gif-sur-Yvette, France

[3]Université Paris-Saclay, CNRS, Institut de Chimie des Substances Naturelles, UPR 2301, 91198, Gif-sur-Yvette, France

[4]Microscopy and Cryo-electron Microscopy Service Group, Max Planck Institute for Molecular Genetics, Ihnestrasse 63-73, 14195, Berlin, Germany.

* Corresponding author : isabelle.auzat@i2bc.paris-saclay.fr

This PDF file includes:

- Supplementary Tables 1 - 3

Supplementary **Table 1** Bacterial strains, bacteriophages and plasmids used in this work. [p2]

Supplementary **Table 2** Single nucleotide changes identified by NGS of SPP1*sus999* (*gp16.1*$^{-}$) DNA relative to the SPP1 wild type reference sequence (X97918.3 [13]). [p3]

Supplementary **Table 3** Genetic origin of mutations in phage SPP1*sus999* (gp16.1^{-}) DNA. [p3]

- Supplementary Figures 1 - 7

Supplementary **Figure 1** Gp16.1 purification and association state. [p4]

Supplementary **Figure 2** Phage purification by CsCl isopycnic centrifugation monitored by EM. [p5]

Supplementary **Figure 3** Thermostability of SPP1*gp16.1*$^{-}$ (gp16.1^{-}) and SPP1*gp16.1*$^{-}$ (gp16.1^{+}) phage particles. [p6]

Supplementary **Figure 4** Ejection of phages SPP1*gp16.1*$^{-}$ (gp16.1^{-}) and SPP1*gp16.1*$^{-}$ (gp16.1^{+}) DNA *in vivo*. [p7]

Supplementary **Figure 5** Sequence homology between experimentally studied TCPs. [p8]

Supplementary **Figure 6** Homology search of TCPs with known function and of THJPs from the same phages. [p9-p31]

- Bacteriophage SPP1 [p10-12]
- Bacteriophage Lambda [p13-15]
- Bacteriophage T5 [p16-18]
- Bacteriophage TP901-1 [p19-21]
- Bacteriophage HK97 [p22-24]
- Bacteriophage P2 [p25-27]
- Bacteriophage Mu [p28-30]
- Legend [p31]

Supplementary **Figure 7** Uncropped and unedited images of X-ray films and gels shown in the main and supplementary figures. [p32]

- Supplementary references [p33]

Supplementary Table 1: bacterial strains, bacteriophages and plasmids used in this work.

Strains, phages and plasmids	Relevant genotype or phenotype	Source or reference
***E. coli* strains**		
DH5α	F^- *Φ80lacZΔM15 Δ(lacZYA-argF) U169 recA1 endA1 hsdR17 (rk-, mk+) phoA supE44 λ-thi-1 gyrA96 relA1*	1<br>Lab. stock
JS218	K-12 [MC1061 derivative (F^-) *araD139* Δ(*ara-leu*)*7696* Δ(*lac*)*X74 galU galK hsdR2 mcrB1 rpsL* (Str^r) *malP*::*$lacI^q$*]	2
BL21 (DE3)	F^-, *omp*T, *hsd*S_B (r_B-, m_B-), *dcm*, *gal*, λ(DE3)	Lab. stock
BL21 (DE3) (pLysS)	F^-, *omp*T, *hsd*S_B (r_B-, m_B-), *dcm*, *gal*, λ(DE3), pLysS, Cm^r.	Lab. stock
***B. subtilis* strains**		
YB886	*amyE trpC2 metB5 xin-1 att*SPβ	3
CSJ4 (Δ*yueB*)	YB886 derivative strain with a deletion covering most of the *yueB* receptor gene and with gene *eryR* inserted in the *amyE* locus	4
HA101B	[*his met*] *sup- leu trpC2*; Suppressor strain	5
***B. subtilis* phages**		
SPP1*wt*	SPP1 wild-type lytic siphophage	6
SPP1*$gp13^-$*	SPP1*sus31* defective in gene *13* coding for the major capsid protein	7
SPP1*$gp16.1^-$-(gp17*)*	SPP1*sus666* defective in gene *16.1* coding for the tail completion protein and with an additional mutation* in gene *17*	8,9, this work
SPP1*$gp16.1^-$*	SPP1*sus999* defective in gene *16.1* coding for the tail completion protein	this work
SPP1*$gp13^-$-$gp16.1^-$*	SPP1*sus31-sus999* defective in genes *13* and *16.1*	this work
SPP1*$gp17^-$*	SPP1*sus82* defective in gene *17* coding for the tail-to-head joining protein	9
SPP1*$gp16.1^-$-$gp17^-$*	SPP1*sus999-sus82* defective in genes *16.1* and *17*	this work
SPP1*$gp17.1^-$*	SPP1*sus45* defective in genes *17.1 coding for the tail tube proteins.*	9,10
SPP1*$gp16.1^-$-$gp17.1^-$*	SPP1*sus999-sus45* defective in genes *16.1* and *17.1* (without the additional mutation in gene *17* found in SPP1*sus666)*	this work
**Plasmids**		
pBT361	A derivative of vector pRSET-A (Invitrogen) coding for 6His-gp16.1.	8
pBT378	A derivative of PHP13 coding for gp13.	11
pIA2	Shuttle vector that replicates in *E. coli* and *B. subtilis* cells. Provides a cloning cassette with an N-term_6His tag.	12
pIA3	Shuttle vector that replicates in *E. coli* and *B. subtilis* cells. Provides a cloning cassette with a C-term_6His tag.	12
pIA19	A derivative of pIA2 coding for 6His-gp16.1.	this work
pIA20	A derivative of pIA3 coding for gp16.1-6His.	this work
pIA21	A derivative of pIA2 coding for 6His-gp17.	12
pIA23	A derivative of pIA2 coding for 6His-gp17.1.	this work
pPT25	A derivative of PHP13 coding for gp16.1, gp17 and gp17.1.	10

Supplementary Table 2: Single nucleotide changes identified by NGS of SPP1*gp16.1*$^-$ DNA relative to the SPP1 wild type reference sequence (X97918.3 [13]).

Position	Reference base	Observed base	Mutation frequency	Coverage	ORF	Triplet mutation	Protein mutation	Protein function
23529	G	A	99,98%	50321	*29.1*	GTT ↓ ATT	Val_{58} ↓ Ile	unknown
27290	C	T	99,99%	53143	*32*	CAC ↓ CAT	His_{671} ↓ His	putative ATP-binding protein
43913	C	T	99,98%	29906	*1*	CCC ↓ CCT	Pro_{10} ↓ Pro	small terminase subunit (TerS)

Supplementary Table 3: Genetic origin of mutations in phage SPP1*sus999* DNA. Three mutations were identified by NGS in phage SPP1*gp16.1*$^-$ DNA at positions 23529 (gene *29.1*), 27290 (gene *32*), and 43913 (gene *1*) (Supplementary Table 2). Each region around these three mutated positions was amplified by PCR and sequenced for the phage SPP1*sus999* stock as well as for the four phage stocks used to construct it (SPP1*sus666*, SPP1*sus45*, SPP1*sus999-sus45* and SPP1*wt* (Supplementary Table 1)). Bases in green correspond to *wt* sequences and those in red to mutated bases in SPP1*gp16.1*$^-$. The mutations at positions 23529 and 27290 come from the parental phage SPP1*sus45*, while the mutation at position 43913 comes from SPP1*sus666*. Preparations of SPP1*sus999* phages with and without gp16.1 produced in parallel for this study were also sequenced, and both show all three mutations in their genome.

Phage cross 1	SPP1*sus666*<br>SPP1*gp16.1*$^-$-(*gp17**)<br>23529 : G<br>27290 : C<br>43913 : T	x	SPP1*sus45*<br>SPP1*gp17.1*$^-$<br>23529 : A<br>27290 : T<br>43913 : C		
		↓			
Phage cross 2		SPP1*sus999-sus45*<br>SPP1*gp16.1*$^-$-*gp17.1*$^-$<br>23529 : A<br>27290 : T<br>43913 : T	x	SPP1*wt*<br><br>23529 : G<br>27290 : C<br>43913 : C	
			↓		
			SPP1*sus999*<br>SPP1*gp16.1*$^-$<br>23529 : A<br>27290 : T<br>43913 : T		

B. subtilis host strain	Permissive strain (HA101B)	Non-permissive strain (YB886)
Phages used in this study for biochemical and EM experiments	SPP1*sus999*<br>SPP1*gp16.1*$^-$<br>(gp16.1$^+$)<br>23529 : A<br>27290 : T<br>43913 : T	SPP1*sus999*<br>SPP1*gp16.1*$^-$<br>(gp16.1$^-$)<br>23529 : A<br>27290 : T<br>43913 : T

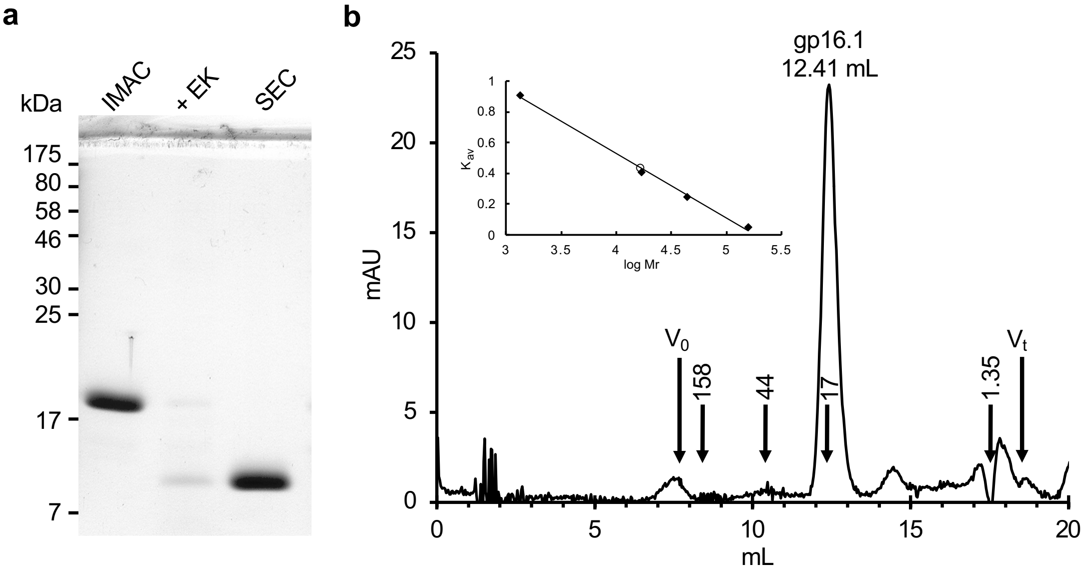

Supplementary Figure 1 | Gp16.1 purification and association state. a Coomassie Blue stained SDS-PAGE of 6His-gp16.1 purified by Immobilized Metal Affinity Chromatography (IMAC), after cleavage with enterokinase (+EK), and after Size Exclusion Chromatography (SEC) of the cleaved protein. **b** Elution chromatogram of enterokinase-cleaved gp16.1 from SEC in an analytical column (Superdex 75 (10/300 GL) GE). The elution position of protein standards, the void volume (V_o) and total volume (V_t) of the column is indicated by vertical arrows. The inset shows the calibration line to determine the K_{av}.

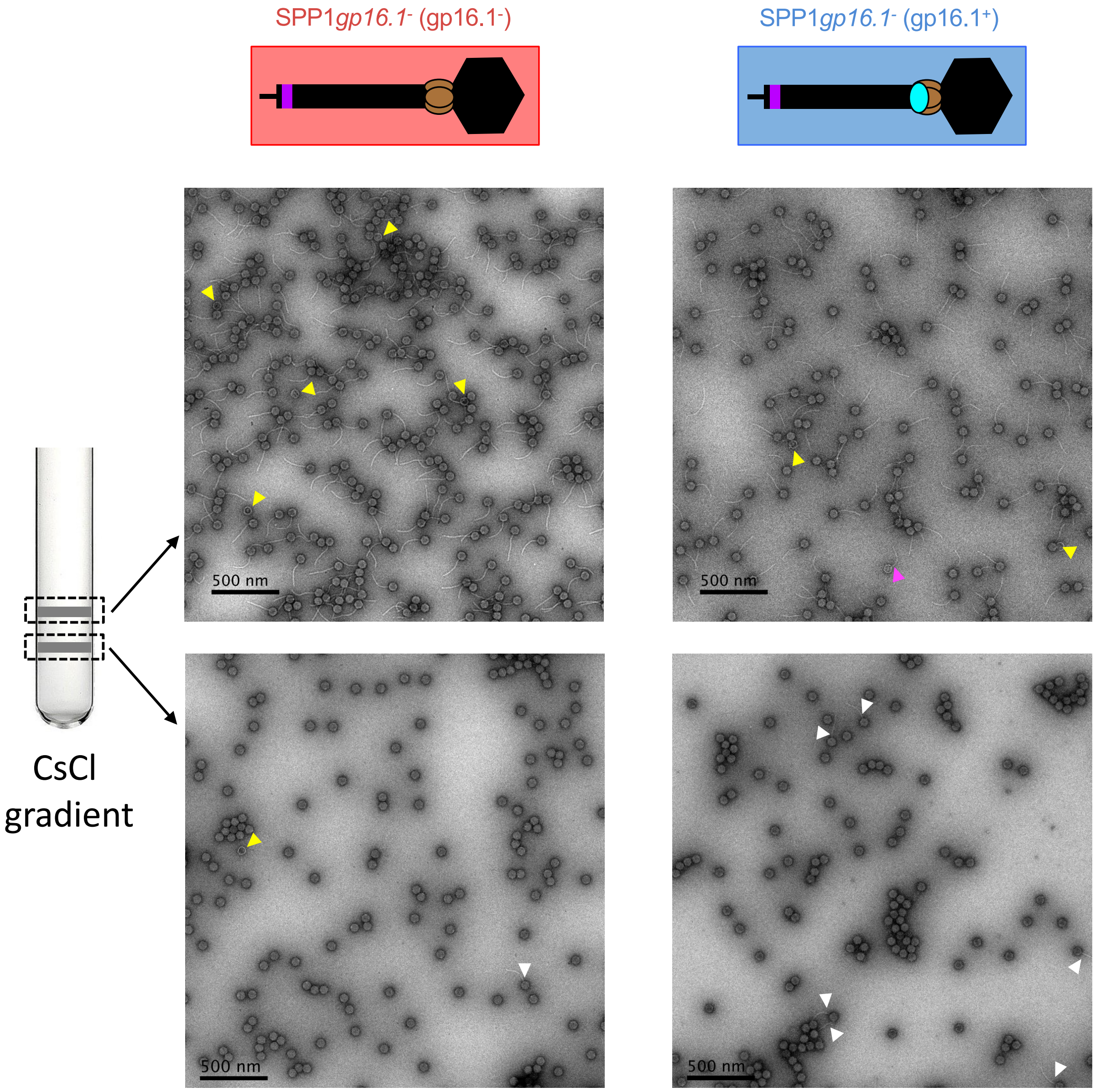

Supplementary Figure 2 | Phage purification by CsCl isopycnic centrifugation monitored by EM. Phages produced during SPP1*gp16.1*$^-$ infection of non-permissive (left) or permissive (right) strains were purified on a discontinuous CsCl gradient. After centrifugation of the lysates, the two visible well-separated bands were collected by puncturing the tube with a syringe, negatively stained and observed by EM. The upper band corresponds to entire phage particles and the lower band to tailless capsids filled with DNA. Colored arrowheads represent rare contaminants: yellow: empty capsids; magenta: empty phage particle; and white: entire phage particles in the bottom band.

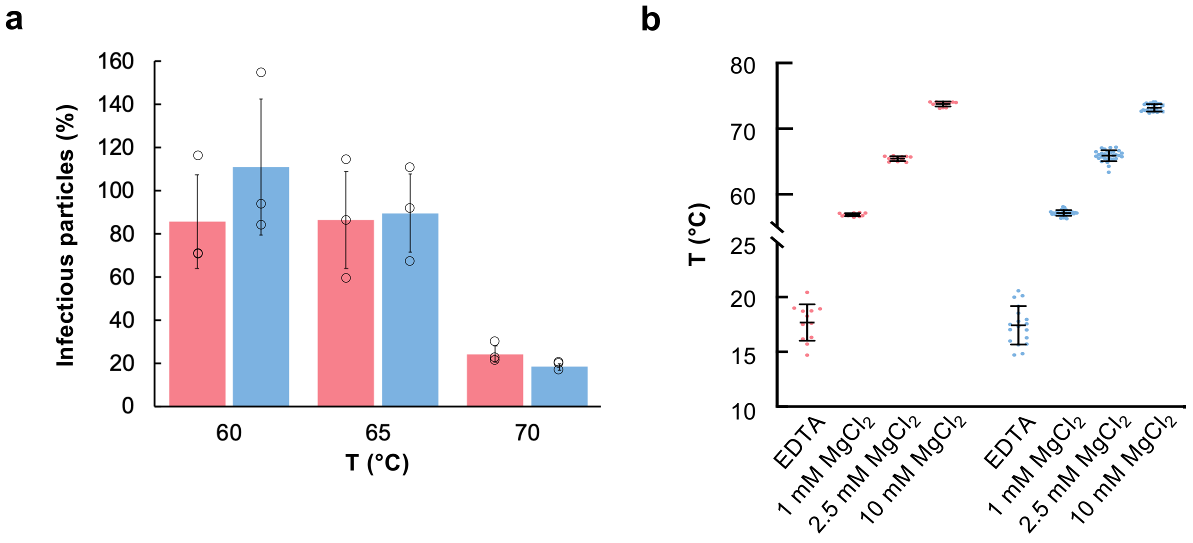

Supplementary Figure 3 | Thermostability of SPP1*gp16.1*⁻ (gp16.1⁻) and SPP1*gp16.1*⁻ (gp16.1⁺) phage particles. a Viability of phage particles without gp16.1 (salmon bars) and with gp16.1 (blue bars) after incubation for 15 min at the temperatures displayed. Phage viability is shown as a percentage of the initial virion input (~10^{11} pfu ml^{-1}), determined from an average of three independent experiments. Empty black circles correspond to experimental points and error bars represent standard deviations. **b** T_m of capsid disruption temperature assessed by DNA release in a Thermal Shift Assay. Phage particles without gp16.1 (salmon dots, left) and with gp16.1 (blue dots, right) were diluted in buffer containing either 10 mM EDTA leading to their physical disruption or increasing concentrations of capsid-stabilizing $MgCl_2$. Error bars represent the standard deviation from at least twelve independent experiments.

a

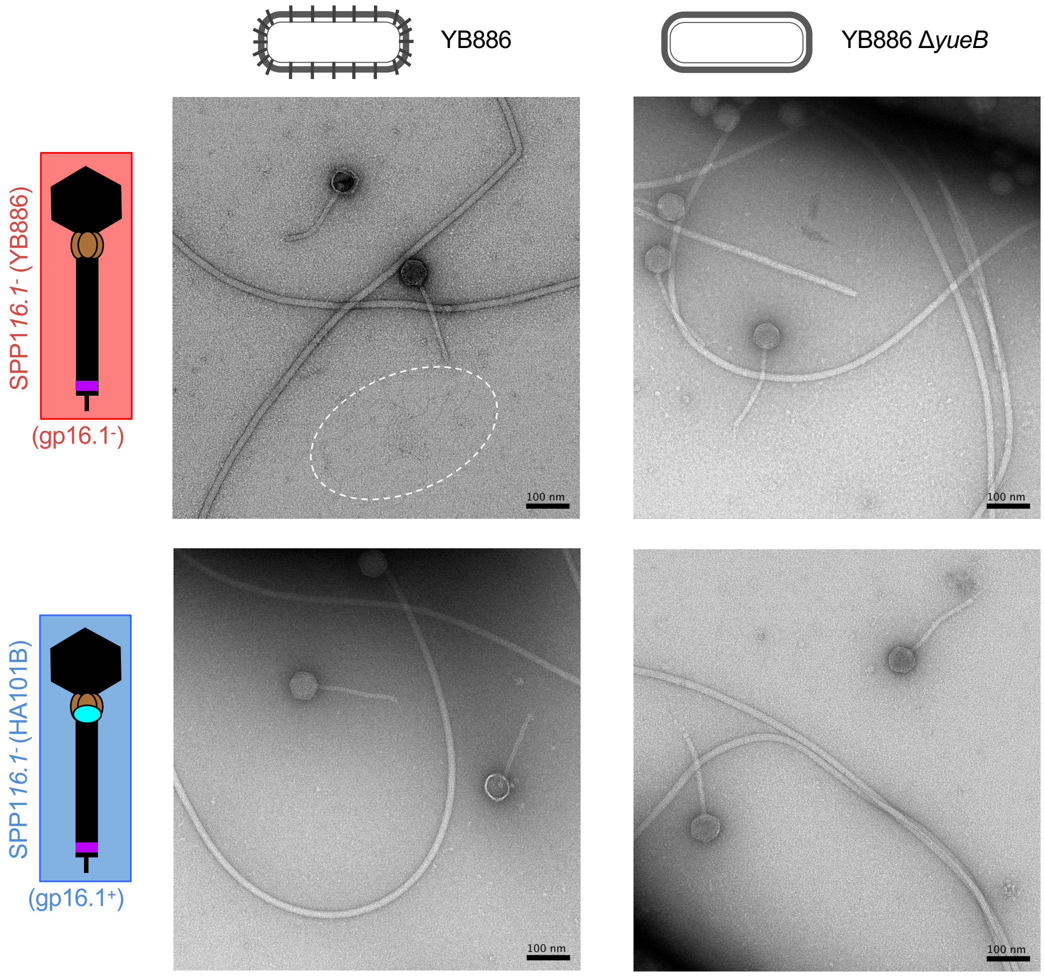

b

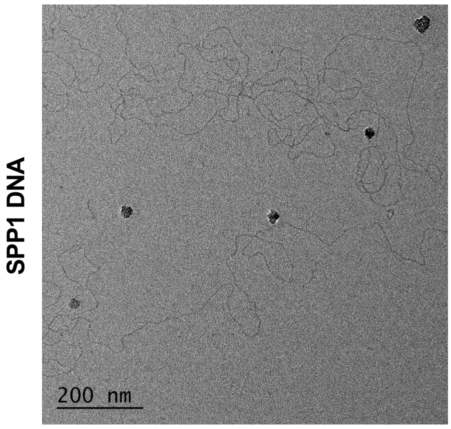

Supplementary Figure 4 | Ejection of phages SPP1*gp16.1*$^{-}$ (gp16.1^{-}) and SPP1*gp16.1*$^{-}$ (gp16.1^{+}) DNA *in vivo*. a Representative electron micrographs of phage-bacterium co-cultures at 5 min post-infection fixed with 1% glutaraldehyde and stained with a low concentration of uranyl acetate (0.2%). The dotted white oval highlights the phage DNA ejected outside the cell. DNA appears positively stained under these experimental conditions. **b** Electron micrograph of purified SPP1 DNA molecule prepared for EM as in **a**.

a

TCPs	SPP1	Lambda	T5	TP901-1	HK97	P2	Mu
SPP1		-	-	1e-11	1e-04	-	-
Lambda	-		-	-	-	-	-
T5	-	-		-	0.013	-	-
Tp901-1	1e-11	-	-		8e-10	-	-
HK97	1e-04	-	0.013	8e-10		-	-
P2	-	-	-	-	-		-
Mu	-	-	-	-	-	-	

b

Query: **SPP1** gp16.1 Length: 144 **/ Tp901-1** ORF40 Length: 112
Score:44.3 bits(103), Expect:1e-11,
Identities:32/115(28%), Positives:46/115(40%), Gaps:27/115(23%)

```
Query  28   LTQVEQVIIKTAEKIAGLAASLAPVDEGNLKNSIQIDYKNNGLTAEITVGAEYAIYVEYG  87
            L  V+QV+      +      L PVD G +K SI+++    G + +     +Y+ YVEYG
Sbjct  23   LKGVQQVVKSNTSNMTANMQKLVPVDTGYMKRSIKMELTEGGFSGQAGPHTDYSAYVEYG  82

Query  88   TGIYAVDGNGRKTPWTYYSPKLGRYVRTQGAPAQPFFWPAVEEGGEYFEREMRRL  142
            T                         R Q   AQPF  PA  E    F +++ RL
Sbjct  83   T-------------------------RFQS--AQPFVKPAYNEQKGVFIKDLERL  110
```

Query: **SPP1** gp16.1 Length: 144 **/ HK97** gp10 Length: 149
Score:25.4 bits(54), Expect:1e-04,
Identities:20/79(25%), Positives:32/79(40%), Gaps:5/79(6%)

```
Query  50   APVDEGNLKNSIQIDYKNNGLTAEITVGAEY-AIYVEYGTGIYAVDGNGRKTPWTYYSPK  108
            APV  G LK ++ +  + +    EI+ G     +    G     +  N  +  + +   +
Sbjct  49   APVRTGKLKKNVVVVTQKSRRRGEISSGVHIRGVNPRTGNSDNTMKANNPRNAFYWRFVE  108

Query  109  LGRYVRTQGAPAQPFFWPA  127
            LG    T   PA PF  PA
Sbjct  109  LG----TANMPAHPFVRPA  123
```

Query: **T5** p143 Length: 255 **/ HK97** gp10 Length: 149
Score:20.4 bits(41), Expect:0.013,
Identities:13/33(39%), Positives:17/33(51%), Gaps:9/33(27%)

```
Query  11  IKEQLDTASRSENNKNTVVYSVETGLKDPTRDG  43
           I + L+  SR+ENNK          L+D TR G
Sbjct  14  IAKDLEALSRAENNK---------VLRDATRAG  37
```

Query: **Tp901-1** ORF40 Length: 112 **/ HK97** gp10 Length: 149
Score:39.7 bits(91), Expect:8e-10,
Identities:29/127(23%), Positives:56/127(44%), Gaps:27/127(21%)

```
Query  1    MKSSLSFKGIDQLVKHLD---KAASLKGVQQVVKSNTSNMTANMQKLVPVDTGYMKRSI-  56
            +++SL F G++ + K L+   +A + K ++   ++    +   +    PV TG +K+++
Sbjct  2    IETSLDFSGLNDIAKDLEALSRAENNKVLRDATRAGAEVLKEEVIDRAPVRTGKLKKNVV  61

Query  57   --------KMELTEGGFSGQAGPHTD---------------YSAYVEYGTRFQSAQPFVK  93
                    + E++ G       P T                Y  +VE GT    A PFV+
Sbjct  62   VVTQKSRRRGEISSGVHIRGVNPRTGNSDNTMKANNPRNAFYWRFVELGTANMPAHPFVR  121

Query  94   PAYNEQK  100
            PAY+ ++
Sbjct  122  PAYDTRE  128
```

Supplementary Figure 5 | Sequence homology between experimentally studied TCPs. a E-values of pairwise sequence alignments between TCPs using pBlast default parameters. (-) indicates no significant similarity found. **b** Output of pairwise alignments between TCPs with sequence homology.

Supplementary Figure 6
(pages 9-31)

Bacteriophage SPP1: TCP and THJP sequences

**TCP**	**gp16.1**	MSVRIDPSWRRIMSRNVRTFSGHVLTQVEQVIIKTAEKIAGLAASLAPVDEGNLKNSIQI<br>DYKNNGLTAEITVGAEYAIYVEYGTGIYAVDGNGRKTPWTYYSPKLGRYVRTQGAPAQPF<br>FWPAVEEGGEYFEREMRRLRG
**THJP**	**gp17**	MTWKLASRALQKATVENLESYQPLMEMVNQVTESPGKDDPYPYVVIGDQSSTPFETKSSF<br>GENITMDFHVWGGTTRAEAQDISSRVLEALTYKPLMFEGFTFVAKKLVLAQVITDTDGVT<br>KHGIIKVRFTINNN

blastp (version: BLASTP 2.12.0+)
Database: uniprotkb_refprotswissprot
Sequence: EMBOSS_001
Length: 141

Mon, Dec 11, 2023 at 10:16:34
Mon, Dec 11, 2023 at 10:17:23

Sequence Match (1–141) | E-value | Subject Match (1–175)

		E-value
O48447_BPSPP	Bacteriophage SPP1 compl...	4.0E-99
A0A5P8PI31_9CAUD	Putative tail protein OS...	1.0E-98
A0A5P8PHN3_9CAUD	Putative tail protein OS...	1.8E-90
A0A5B9NH20_9CAUD	HK97 gp10 family phage p...	1.0E-58
A0A386K763_9CAUD	HK97 gp10 family phage p...	1.6E-52
Q65KN8_BACLD	Phage related protein OS...	1.7E-50
A0A0F5HN16_BACTR	Phage capsid and scaffol...	1.3E-46
A0A385YTB6_9BACL	HK97 gp10 family phage p...	4.4E-44
A0A059NYL1_9BACI	Phage protein, HK97 gp10...	2.4E-42
A0A2W7MVR7_9BACI	HK97 gp10 family phage p...	3.2E-40
A0A2S5D0B2_LYSSH	HK97 gp10 family phage p...	2.4E-39
A0A0X1RWF1_9BACL	Bacteriophage HK97-gp10,...	3.3E-37
A0A0U2ZCI3_9BACL	HK97 gp10 family phage p...	7.6E-37
A0A0M4FTY1_9BACI	HK97 gp10 family phage p...	4.5E-36
A0A0C2VQ67_9BACL	HK97 gp10 family phage p...	1.3E-32
A0A0C2VMU2_9BACL	HK97 gp10 family phage p...	3.5E-32
A0A2S5CZK3_LYSSH	HK97 gp10 family phage p...	1.3E-29
A0A0E3T8J0_9CAUD	Putative tail-component ...	7.6E-25
A0A3T0IIJ0_9CAUD	Putative tail-component ...	7.6E-25
M9NRJ3_9CAUD	HK97 gp10 family phage p...	4.6E-24
A0A059T7Q2_9CAUD	Structural protein OS=St...	1.2E-23
A0A482MG97_9CAUD	Putative tail component ...	1.2E-23
W5R9L6_9CAUD	Putative major tail prot...	1.4E-23
A9CRB4_BPMR1	Major tail protein OS=St...	1.4E-23
A0A7U0G7U7_9CAUD	Capsid and scaffold prot...	1.8E-23
Q9FZZ7_9CAUD	Uncharacterized protein ...	2.0E-23
A0A0H4IPA6_9CAUD	Tail protein OS=Staphylo...	2.6E-23
Q4ZBB3_9CAUD	ORF041 OS=Staphylococcus...	2.6E-23
A7YGV1_9CAUD	HK97 gp10 family phage p...	3.6E-23
A0A0E3XCY8_9CAUD	Putative tail-component ...	3.6E-23
A0A9E7ML03_9CAUD	Capsid and scaffold prot...	1.0E-22
A0A6C0R367_9CAUD	Capsid and scaffold prot...	1.0E-22
R4IG77_9CAUD	HK97 gp10 family phage p...	1.0E-22
Q4ZCE4_9CAUD	ORF040 OS=Staphylococcus...	1.0E-22
Q4ZC65_9CAUD	ORF033 OS=Staphylococcus...	1.4E-22
Q4L3W8_STAHJ	HK97 gp10 family phage p...	2.1E-22
A0A7D7EXC9_9CAUD	HK97 gp10 family phage p...	2.1E-22
A0A2I6PCV6_9CAUD	HK97 gp10 family phage p...	1.2E-21
A0A7U0GDJ4_9CAUD	Tail complementation pro...	3.3E-21
A0A7U0GDX7_9CAUD	Tail complementation pro...	3.3E-21
A0A7U0GDU0_9CAUD	Tail complementation pro...	3.3E-21
A0A1W6JP98_9CAUD	Capsid and scaffold prot...	3.3E-21
I6TJP9_9CAUD	HK97 gp10 family phage p...	3.3E-21
A0A7T1JPX5_9CAUD	Capsid and scaffold prot...	4.7E-21
A0A0N9BAY5_9CAUD	Putative head-tail compo...	4.7E-21
A1KXA1_9CAUD	HK97 gp10 family phage p...	6.4E-21
A0A4Y5NY27_9CAUD	HK97 gp10 family phage p...	1.3E-20
I6SB49_9CAUD	HK97 gp10 family phage p...	1.3E-20
A0A0S2MVE4_9CAUD	HK97 gp10 family phage p...	1.8E-20
I7ILS4_9CAUD	Phage protein, HK97 gp10...	1.8E-20
A0A068EM37_9CAUD	HK97 gp10 family phage p...	1.8E-20
A0A410T4R3_9CAUD	Capsid protein OS=Staphy...	2.3E-20
A0A141VTN0_9CAUD	Capsid and scaffold prot...	5.3E-20
A0A0A7AQU9_9CAUD	HK97 gp10 family phage p...	8.0E-19
A0A0H3U4T2_9CAUD	Tail protein OS=Staphylo...	3.5E-18
A0A810PUP6_9FIRM	HK97 gp10 family phage p...	1.2E-17
A0A0C2HK41_9STAP	HK97 gp10 family phage p...	8.5E-17
A0A1S5SAB8_9CAUD	Minor capsid protein OS=...	8.9E-15
A0A4D6AQQ6_9CAUD	Capsid and scaffold prot...	1.2E-14
A0A1S5SAL3_9CAUD	Minor capsid protein OS=...	1.3E-14
A0A1S5S8X0_9CAUD	Minor capsid protein OS=...	1.8E-14
A0A1S5SA22_9CAUD	Minor capsid protein OS=...	1.8E-14
A0A4D5ZY71_9CAUD	Capsid and scaffold prot...	2.4E-14
A0A4D7AJK9_9FIRM	HK97 gp10 family phage p...	3.4E-14
A0A1S5SAK2_9CAUD	Minor capsid protein OS=...	7.0E-14
A0A1D7XLV1_9CLOT	HK97 gp10 family phage p...	4.0E-13
A0Q0U1_CLONN	HK97 gp10 family phage p...	5.1E-13
A0A1W1X024_9CLOT	Phage protein, HK97 gp10...	5.3E-13
A0A8J6IF52_9FIRM	HK97 gp10 family phage p...	8.7E-13
A0A6M0R8D4_9CLOT	HK97 gp10 family phage p...	2.8E-12
A0A098MF11_9BACL	HK97 gp10 family phage p...	3.0E-12
W8EBB9_9CAUD	Tail component protein O...	3.6E-12
A0A1U6JHW4_9CLOT	HK97 gp10 family phage p...	1.2E-11
R5ZK28_9FIRM	HK97 gp10 family phage p...	3.6E-11
G5K3R3_9STRE	Phage protein, HK97 gp10...	6.4E-11
A0A1I3I6P1_9ACTN	Phage protein, HK97 gp10...	9.5E-11
A0A091C7R0_9ENTE	Putative phage tail comp...	1.9E-10
A0A1H0M8C1_9CLOT	HK97 gp10 family phage p...	1.9E-10
A0A1I2NN29_9CLOT	Phage protein, HK97 gp10...	2.0E-10
A0A136Q8F4_9FIRM	Phage protein, HK97 gp10...	3.1E-10
A0A2T4Z923_9BACL	HK97 gp10 family phage p...	3.3E-10
A0A4U0NMF9_9ACTN	HK97 gp10 family phage p...	3.4E-10
A0A7M2RKI7_9FIRM	HK97 gp10 family phage p...	4.2E-10
A0A1M6L1A6_9CLOT	Phage protein, HK97 gp10...	5.4E-10
R4K515_CLOPA	Phage protein, HK97 gp10...	6.0E-10
A0A1Y4UIX9_9FIRM	HK97 gp10 family phage p...	6.1E-10
A0A2M9M5V5_9ACTN	HK97 gp10 family phage p...	6.8E-10
A0A7G9GPM9_9FIRM	HK97 gp10 family phage p...	7.5E-10
A0A9E7P592_9CAUD	HK97 gp10 family phage p...	7.9E-10
A0A3A9EIF3_9FIRM	HK97 gp10 family phage p...	1.3E-9
A0A4D6B0G7_9CAUD	Uncharacterized protein ...	1.4E-9
A0A4D6AXY6_9CAUD	Capsid and scaffold prot...	2.0E-9
R5R6A9_9FIRM	HK97 gp10 family phage p...	2.0E-9
A0A0J8DBB1_CLOCY	Phage protein, HK97 gp10...	4.0E-9
A0A975SBC7_9BACL	HK97 gp10 family phage p...	5.7E-9
A0A4D6A383_9CAUD	Capsid and scaffold prot...	7.7E-9
A0A173W5Z4_9FIRM	Phage protein, HK97 gp10...	8.3E-9
R6RKC5_9FIRM	HK97 gp10 family phage p...	1.9E-8
A0A249XNM6_9CAUD	Minor tail protein OS=Br...	4.0E-8
A0A1Q4W4C0_9ACTN	HK97 gp10 family phage p...	4.0E-8
R9QNC6_9CAUD	Putative activator of ta...	4.5E-8
A0A649V1X0_9CAUD	Capsid and scaffold prot...	4.5E-8
A0A1P8BLE3_9CAUD	Activator of tail termin...	4.5E-8
A0A974BIG6_SEDHY	HK97 gp10 family phage p...	4.6E-8
A0A927GY72_9BACL	HK97 gp10 family phage p...	5.1E-8
A0A0F0HPL0_9PSEU	HK97 gp10 family phage p...	6.7E-8
A0A396QET3_9FIRM	HK97 gp10 family phage p...	7.1E-8
A0A653YNT6_9ACTN	Putative Bacteriophage H...	8.1E-8
A0A926IFN3_9FIRM	HK97 gp10 family phage p...	1.2E-7
A0A7X5KS36_9FIRM	HK97 gp10 family phage p...	1.7E-7
R9N6N4_9FIRM	HK97 gp10 family phage p...	1.9E-7
A0A1G8RTT4_9BACI	Phage protein, HK97 gp10...	2.0E-7
A0A367YR52_9ACTN	HK97 gp10 family phage p...	2.1E-7
Q838U0_ENTFA	HK97 gp10 family phage p...	3.7E-7
D2J065_9CAUD	Uncharacterized protein ...	3.7E-7
A0A1M6EXE3_9CLOT	Phage protein, HK97 gp10...	4.9E-7
A0A0R2BLN4_9LACO	HK97 gp10 family phage p...	5.1E-7
A0A2H5YVW2_UNCXX	HK97 gp10 family phage p...	7.4E-7
R5BTF9_9FIRM	HK97 gp10 family phage p...	7.5E-7
A0A7G9WJZ3_9FIRM	HK97 gp10 family phage p...	8.3E-7
A0A7V7QJP8_9FIRM	HK97 gp10 family phage p...	9.5E-7
A0A1Y3TGC1_9FIRM	HK97 gp10 family phage p...	1.2E-6
A0A4D6BEQ0_9CAUD	HK97 gp10 family phage p...	1.5E-6
A0A4D6A7T4_9CAUD	Minor capsid protein OS=...	1.9E-6
A0A7H0XC39_9CAUD	Phage protein, HK97 gp10...	2.0E-6
A0A1Q9JQ38_9FIRM	Phage protein, HK97 gp10...	2.1E-6
A0A1I3UI99_9BACL	Phage protein, HK97 gp10...	2.6E-6
A0A1I2TIM1_9FIRM	Bacteriophage HK97-gp10,...	2.6E-6
A0A2T7BBN1_9BACT	HK97 gp10 family phage p...	2.7E-6
A0A391P8I3_9FIRM	Phage protein OS=Mediter...	2.9E-6
A0A7L5ZKE0_9ACTN	HK97 gp10 family phage p...	3.0E-6
A0A4D6APV6_9CAUD	HK97 gp10 family phage p...	4.1E-6
A0A7M2RFB3_9FIRM	HK97 gp10 family phage p...	4.2E-6
A0A9E7T4U5_9VIRU	HK97 gp10 family phage p...	4.7E-6
N9Y1K6_9CLOT	HK97 gp10 family phage p...	4.9E-6
A0A367FP19_9ACTN	HK97 gp10 family phage p...	7.5E-6
A0A7Y5XM88_9ACTN	HK97 gp10 family phage p...	8.1E-6
A0A0U5J8K6_9CLOT	Phage protein, HK97 gp10...	8.2E-6
A0A6H9YLG9_9ACTN	HK97 gp10 family phage p...	8.3E-6
A0A2I8VH51_9EURY	HK97 gp10 family phage p...	9.2E-6
R6IVT6_9FIRM	Bacteriophage protein PF...	9.9E-6

E-value: 4E-99 — 1.99E-52 — 4.439E-29 — 2.096E-17 — 9.9E-6

European Bioinformatics Institute 2006-2020. EBI is an Outstation of the European Molecular Biology Laboratory.

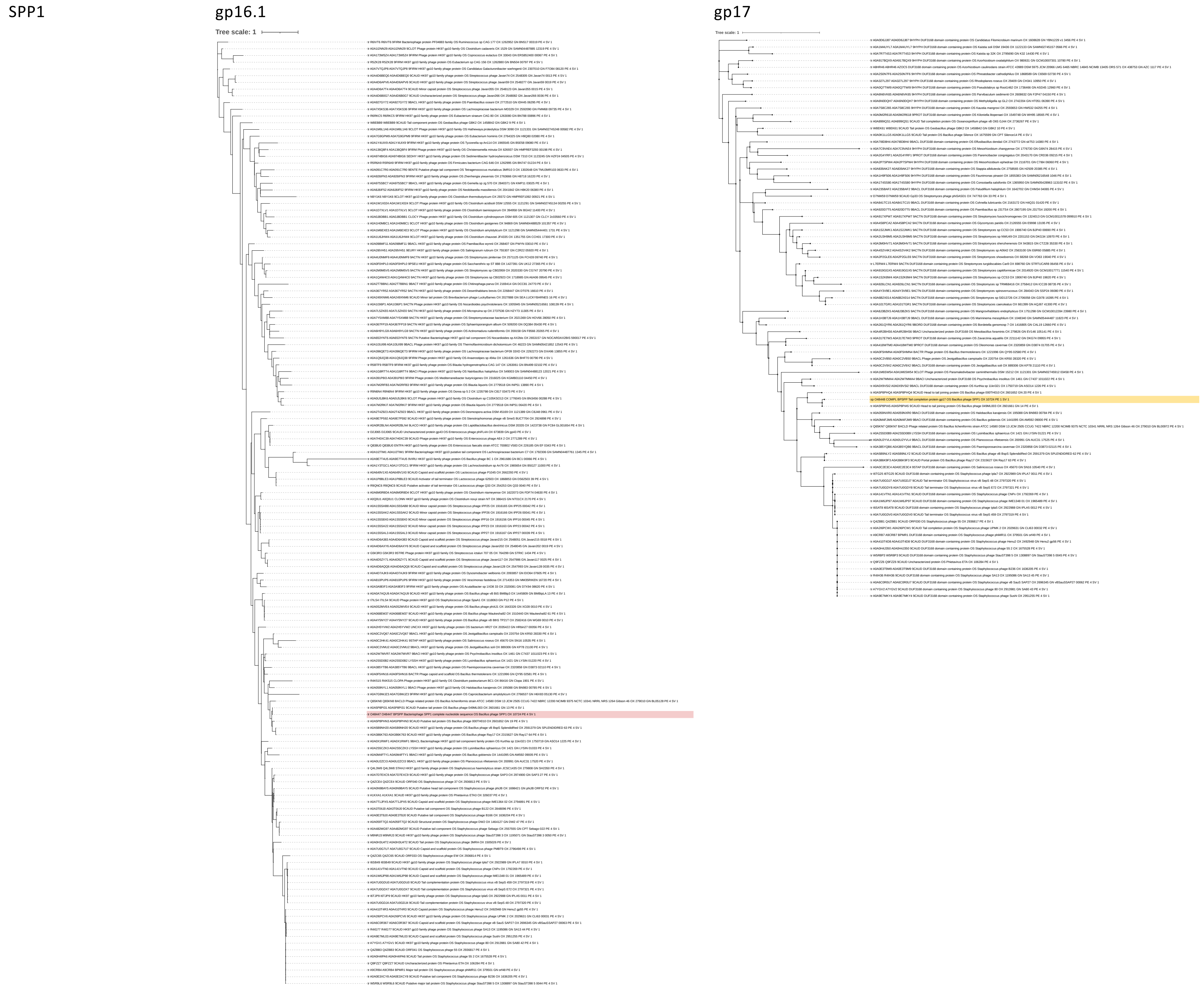
SPP1
gp16.1
Tree scale: 1
gp17
Tree scale: 1

Bacteriophage Lambda: TCP and THJP sequences

TCP **gpZ**

MAIKGLEQAVENLSRISKTAVPGAAAMAINRVASSAISQSASQVARETKVRRKLVKERAR
LKRATVKNPQARIKVNRGDLPVIKLGNARVVLSRRRRRKKGQRSSLKGGGSVLVVGNRRI
PGAFIQQLKNGRWHVMQRVAGKNRYPIDVVKIPMAVPLTTAFKQNIERIRRERLPKELGY
ALQHQLRMVIKR

THJP **gpU**

MKHTELRAAVLDALEKHDTGATFFDGRPAVFDEADFPAVAVYLTGAEYTGEELDSDTWQA
ELHIEVFLPAQVPDSELDAWMESRIYPVMSDIPALSDLITSMVASGYDYRRDDDAGLWSS
ADLTYVITYEM

blastp (version: BLASTP 2.12.0+)

Mon, Dec 11, 2023 at 10:16:53
Mon, Dec 11, 2023 at 10:17:33

Database: uniprotkb_refprotswissprot
Sequence: EMBOSS_001
Length: 192

	Sequence Match (1–192)	E-value
COMPL_LAMBD	Tail completion protein ...	5.5E-128
A0A7Z1AD62_9GAMM	Prophage minor tail prot...	5.5E-128
A0A1S1V5Y2_9FIRM	Prophage minor tail prot...	5.5E-128
A0A417Y9I6_9BACI	Phage tail protein OS=Oc...	5.5E-128
V5BNZ1_9GAMM	Minor tail protein Z OS=...	5.5E-128
A0A021X5X7_9HYPH	Minor tail protein Z OS=...	5.5E-128
A0A083ZYS8_9GAMM	Prophage minor tail prot...	5.5E-128
A0A140NCA8_ECOBD	Prophage minor tail Z fa...	5.5E-128
A0A939DTV0_9ALTE	Phage tail protein OS=Bo...	5.5E-128
A0A346FJ29_9CAUD	Minor tail fiber protein...	5.5E-128
A0A2R9YJK4_9CAUD	Prophage minor tail Z fa...	5.5E-128
K7P6Q6_9CAUD	Minor tail protein Z OS=...	5.5E-128
K7PHR7_9CAUD	Minor tail protein OS=Es...	5.5E-128
A0A7G0Y974_9CAUD	Tail component OS=Escher...	5.5E-128
A0A0K2FI48_9CAUD	Tail component OS=Escher...	5.5E-128
B7LBC5_ECO55	Minor tail protein Z (Gp...	1.6E-127
A0A0B8XLK5_9SPHI	Prophage minor tail prot...	1.6E-127
A0A653FTF1_9CAUD	Phage Ne1 neck protein O...	2.2E-127
Q3YZJ9_SHISS	Tail component of propha...	3.2E-127
A0A143ENV1_ECO57	Phage minor tail protein...	4.5E-127
A0A0H2V9X3_ECOL6	Putative tail fiber comp...	6.4E-127
A0A653FRU2_9CAUD	Phage Ne1 neck protein O...	6.4E-127
A0A653FTY7_9CAUD	Phage Ne1 neck protein O...	6.4E-127
A0A653FR92_9CAUD	Phage Ne1 neck protein O...	1.8E-126
A0A0H2YYM9_ECOK1	Tail fiber component Z o...	1.8E-126
A0A0H2V6W8_ECOL6	Putative tail fiber comp...	1.8E-126
A0A653FVG2_9CAUD	Phage Ne1 neck protein O...	1.8E-126
A0A653FTA3_9CAUD	Phage Ne1 neck protein O...	1.8E-126
A0A653FTE4_9CAUD	Phage Ne1 neck protein O...	1.8E-126
A0A653FRB1_9CAUD	Phage Ne1 neck protein O...	2.6E-126
A0A0H3EFZ8_ECO8N	Putative minor tail prot...	5.3E-126
A0A653FSF3_9CAUD	Phage Ne1 neck protein O...	1.8E-124
A0A653FRG8_9CAUD	Phage Ne1 neck protein O...	1.8E-124
B7MEV5_ECO45	Minor tail protein Z (Gp...	1.9E-124
A0A3N9TAN3_9VIBR	Phage tail protein (Frag...	3.6E-103
A0A653FQD2_9CAUD	Phage Ne1 neck protein O...	3.2E-87
A0A0H2YY69_ECOK1	Minor tail protein OS=Es...	6.8E-82
B2U0C9_SHIB3	Prophage minor tail prot...	6.8E-82
B7ML55_ECO45	Minor tail protein Z (Gp...	6.8E-82
B2TZ71_SHIB3	Prophage minor tail prot...	7.9E-81
A0A152GJS4_ECO57	Phage minor tail protein...	2.2E-80
B7ULV0_ECO27	Predicted minor tail pro...	2.6E-79
A0A0H2V6I2_ECOL6	Putative tail component ...	3.5E-77
B2TW02_SHIB3	Prophage minor tail prot...	4.8E-71
K7P7A8_9CAUD	Minor tail protein Z OS=...	1.8E-69
K7PKQ5_9CAUD	Minor tail protein Z OS=...	1.8E-69
COMPL_BPN15	Tail completion protein ...	3.6E-69
B7L473_ECO55	Minor tail protein Z (Gp...	3.0E-67
A0A485ABE1_KLUCR	Prophage minor tail prot...	9.7E-67
H5UYW4_ATLHE	Putative prophage minor ...	4.5E-65
B7LET5_ECO55	Z protein OS=Escherichia...	5.0E-65
E3G2U5_ENTLS	Prophage minor tail Z fa...	3.7E-64
S5MBY4_9CAUD	Minor tail protein OS=Es...	1.1E-63
A0A7R7D0S8_9CAUD	Phage minor tail protein...	1.1E-63
A0A7U3LQC8_9CAUD	Phage minor tail protein...	1.1E-63
A0A9E7LRT2_9CAUD	Tail component Z OS=Esch...	2.3E-63
A0A0H3JGA8_ECO57	Phage minor tail protein...	1.8E-62
A0A7R7D2G0_9CAUD	Phage minor tail protein...	1.8E-62
Q9EYD6_BPVT1	Minor tail protein Z OS=...	1.8E-62
A0A7R7D2N7_9CAUD	Phage minor tail protein...	1.8E-62
D4E0Q4_SEROD	Prophage minor tail prot...	4.2E-54
M9NZH5_BPPH8	Minor tail protein OS=En...	6.0E-53
A0A1B7JGQ6_9GAMM	Minor tail protein Z OS=...	2.6E-52
A0A873WPA6_9CAUD	Tail component Z OS=Prov...	5.0E-52
K7PH83_9CAUD	Minor tail protein OS=En...	6.5E-52
K7PMB7_9CAUD	Minor tail protein Z OS=...	6.5E-52
Q8ZQG7_SALTY	Fels-1 phage tail compon...	3.9E-51
Q8ZN01_SALTY	Gifsy-1 prophage OS=Salm...	5.2E-51
B7L8P6_ECO55	Minor tail protein Z (GP...	2.1E-50
B7UNW0_ECO27	Predicted minor tail pro...	2.1E-50
K7PKI6_9CAUD	Minor tail protein OS=En...	2.1E-50
A0A9E7E204_9CAUD	Tail component Z OS=Esch...	2.1E-50
A5LH33_9CAUD	Putative tail component ...	2.1E-50
A0A653FS33_9CAUD	Ne1 neck protein OS=Esch...	6.0E-50
A0A291AWZ0_9CAUD	Putative minor tail prot...	8.5E-50
A0A653FTB6_9CAUD	Ne1 neck protein OS=Esch...	1.2E-49
A0A0H2VVL3_SHIFL	Tail component of propha...	3.6E-49
A0A0H2Z6Y6_ECOK1	Tail component of propha...	4.9E-49
B7MNG1_ECO45	Minor tail protein Z (GP...	4.9E-49
A0A9E7J794_9CAUD	Tail component Z OS=Esch...	4.9E-49
A0A0J5KPM7_PLUGE	Tail protein OS=Pluralib...	9.4E-49
Q8X5G0_ECO57	Phage minor tail protein...	9.7E-49
A0A0L0ARH6_9ENTR	Tail protein OS=Klebsiel...	5.4E-48
A0A0F0VDB1_PLUGE	Tail protein OS=Pluralib...	2.0E-45
A0A976SMU6_9CAUD	Tail completion protein ...	2.2E-43
A0A7L5GFN9_9CAUD	Minor tail protein OS=Vi...	3.2E-43
A0A0H3EH24_ECO8N	Putative tail component ...	3.7E-36
Q83R61_SHIFL	Tail component of propha...	1.5E-35
B8F4K4_GLAP5	Putative phage tail comp...	1.4E-25
A0A7W2EXG7_9BURK	Phage tail protein (Frag...	2.0E-22
A0A4R5TLE6_9MICC	Phage tail protein (Frag...	2.0E-22
A0A4R5PUW2_9PROT	Phage tail protein (Frag...	2.0E-22
A0A6L5E3Z9_9BACT	Phage tail protein (Frag...	2.0E-22
A3MZN1_ACTP2	Putative phage tail comp...	3.5E-22
A0A448K0H3_PASAE	Putative tail component ...	9.5E-22
Q8ZQ91_SALTY	Gifsy-2 prophage probabl...	2.9E-20
A0A2U0TAF2_9PAST	Minor tail protein Z (GP...	9.0E-19
A0A085G107_9GAMM	Minor tail protein Z OS=...	3.2E-14
A0A7V8QM55_9GAMM	Phage tail protein (Frag...	5.7E-12
A0A919XJ27_9BACL	Phage tail protein OS=Pa...	2.1E-6

Subject Match (1–214)

E-value: 5.5E-128 | 3.399E-67 | 8.448E-37 | 1.332E-21 | 2.1E-6

European Bioinformatics Institute 2006-2020. EBI is an Outstation of the European Molecular Biology Laboratory.

Lambda

gpZ

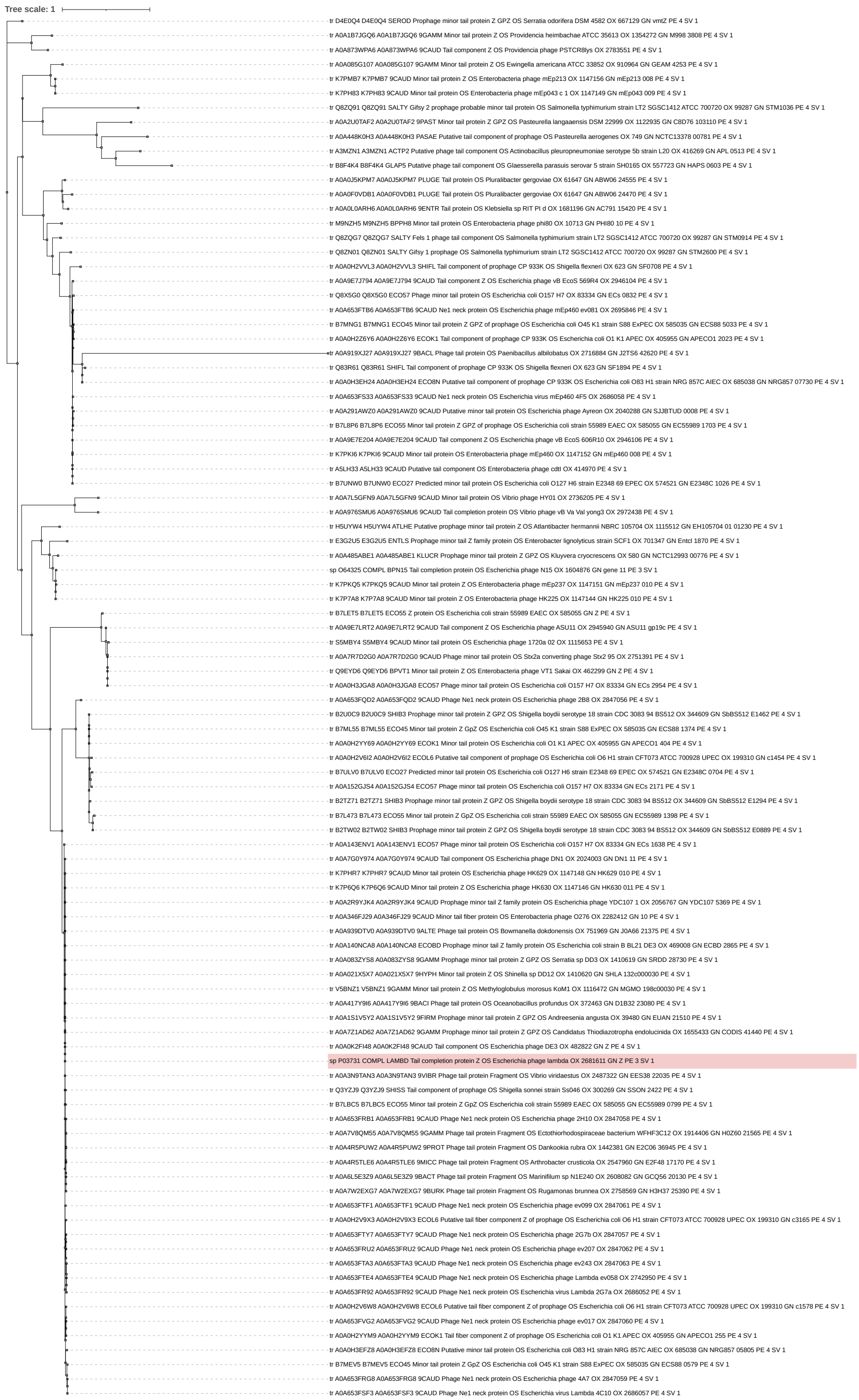

gpU

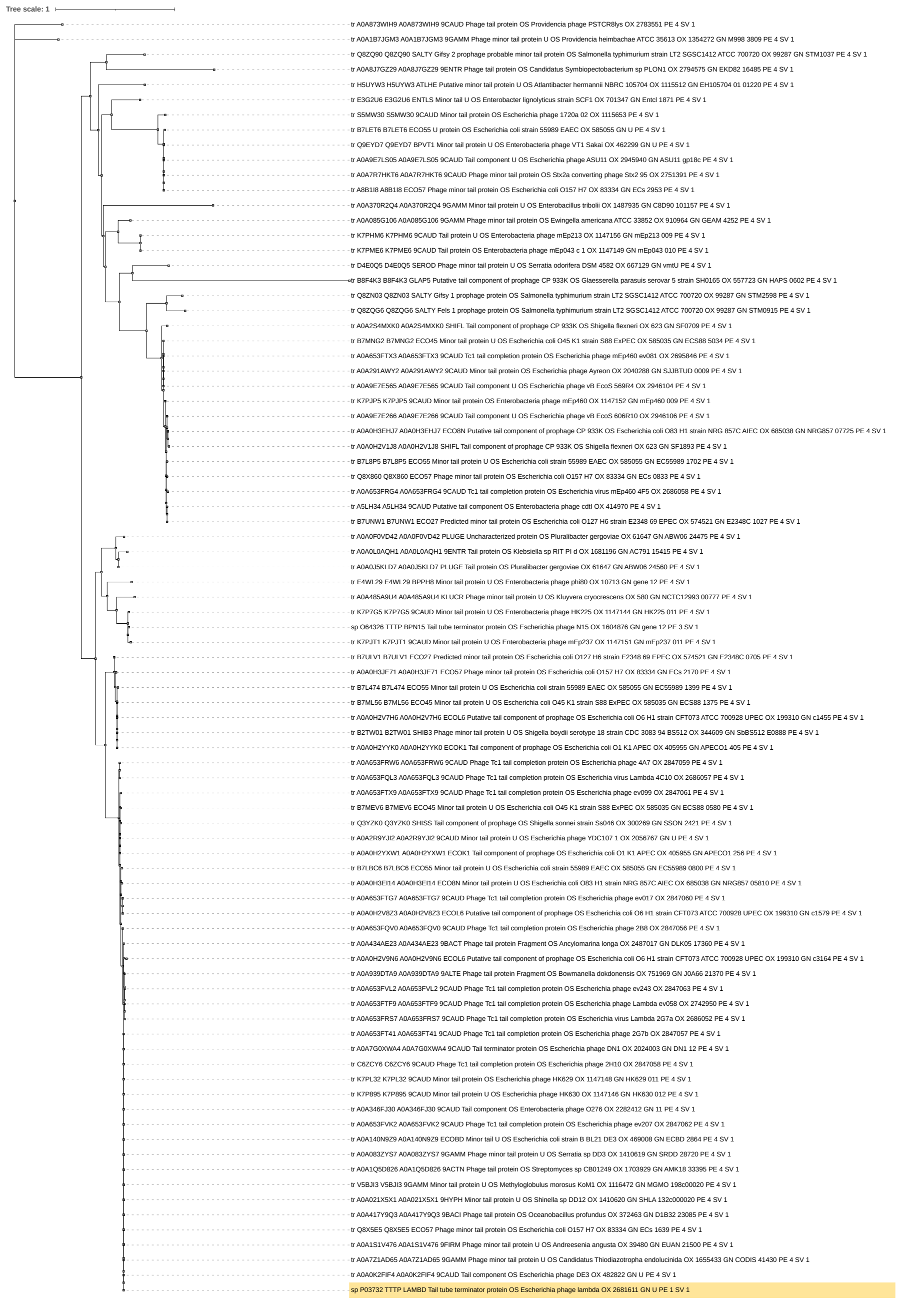

Bacteriophage T5: TCP and THJP sequences

TCP **p143**

MSLSDLARQIIKEQLDTASRSENNKNTVVYSVETGLKDPTRDGTVAQVSFKFSKPVSQDL
LNIRTASILKAVSSSLDLSGDLGALENLIQATAGKKSSVGKKRSTGRVQVNFGDPSDVED
GYSGAVTGASGRFVSNSNMKIILEIVAKEYLIKDMKKAGAPLKFRTGRFANSLKIKDVML
RDSETSKGSPELNVTYNYMTRPYSVFNPAVSTYRRLSLRPYPGARNPQKLIGEAIAKAAR
DLIHSRYKIKVNQGT

THJP **p142**

MDHRTSIAQAMVDRISKQMDGSQPDEYFNNLYGNVSRQTYKFEEIREFPYVAVHIGTETG
QYLPSGQQWMFLELPILVYDKEKTDIQEQLEKLVADIKTVIDTGGNLEYTVSKPNGSTFP
CEATDMIITSVSTDEGLLAPYGLAEINVTVRYQPPRRSLRR

blastp (version: BLASTP 2.12.0+)
Database: uniprotkb_refprotswissprot
Sequence: EMBOSS_001
Length: 255

Mon, Dec 11, 2023 at 10:15:04
Mon, Dec 11, 2023 at 10:15:44

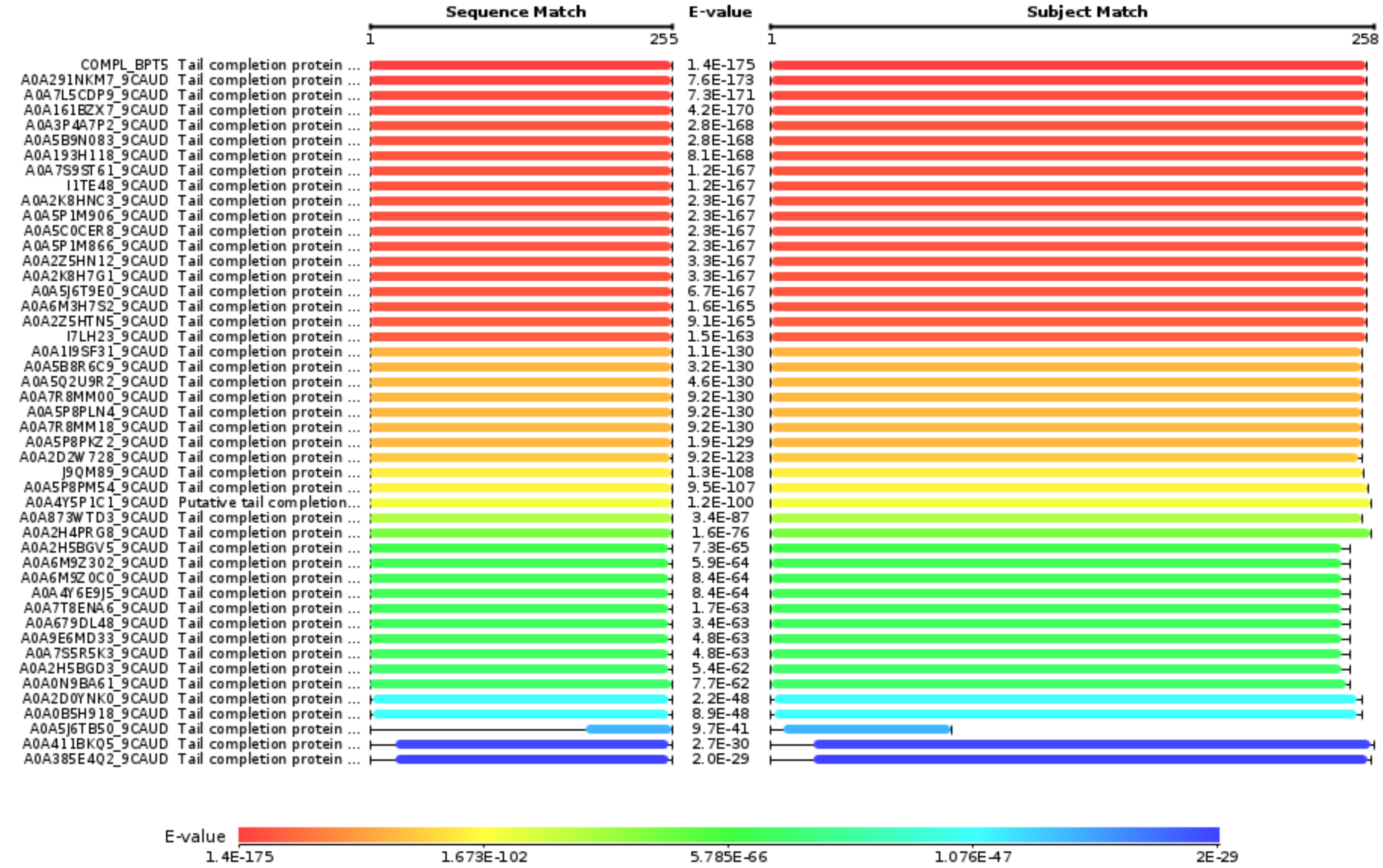

European Bioinformatics Institute 2006-2020. EBI is an Outstation of the European Molecular Biology Laboratory.

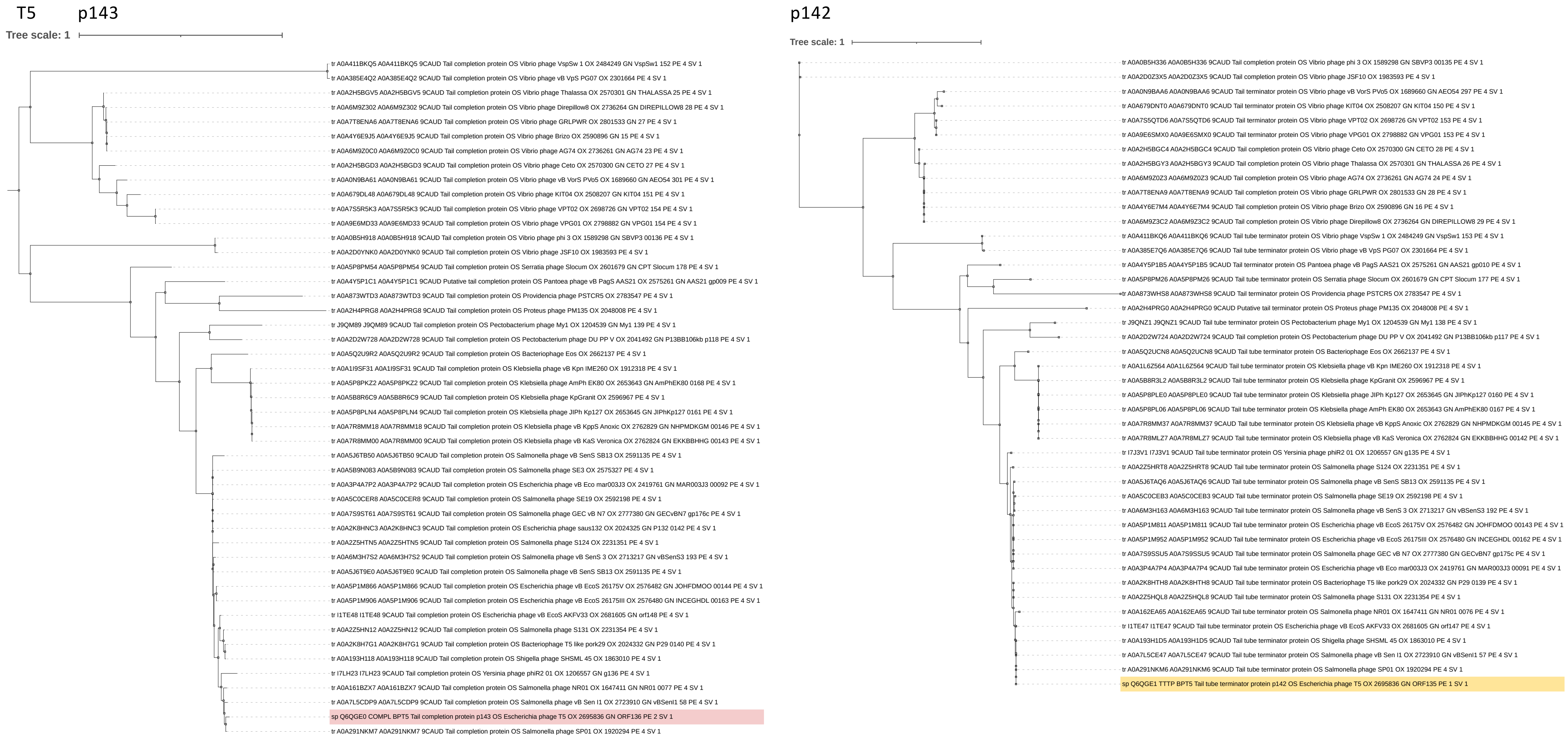

T5
p143
Tree scale: 1
tr A0A411BKQ5 A0A411BKQ5 9CAUD Tail completion protein OS Vibrio phage VspSw 1 OX 2484249 GN VspSw1 152 PE 4 SV 1
tr A0A385E4Q2 A0A385E4Q2 9CAUD Tail completion protein OS Vibrio phage vB VpS PG07 OX 2301664 PE 4 SV 1
tr A0A2H5BGV5 A0A2H5BGV5 9CAUD Tail completion protein OS Vibrio phage Thalassa OX 2570301 GN THALASSA 25 PE 4 SV 1
tr A0A6M9Z302 A0A6M9Z302 9CAUD Tail completion protein OS Vibrio phage Direpillow8 OX 2736264 GN DIREPILLOW8 28 PE 4 SV 1
tr A0A7T8ENA6 A0A7T8ENA6 9CAUD Tail completion protein OS Vibrio phage GRLPWR OX 2801533 GN 27 PE 4 SV 1
tr A0A4Y6E9J5 A0A4Y6E9J5 9CAUD Tail completion protein OS Vibrio phage Brizo OX 2590896 GN 15 PE 4 SV 1
tr A0A6M9Z0C0 A0A6M9Z0C0 9CAUD Tail completion protein OS Vibrio phage AG74 OX 2736261 GN AG74 23 PE 4 SV 1
tr A0A2H5BGD3 A0A2H5BGD3 9CAUD Tail completion protein OS Vibrio phage Ceto OX 2570300 GN CETO 27 PE 4 SV 1
tr A0A0N9BA61 A0A0N9BA61 9CAUD Tail completion protein OS Vibrio phage vB VorS PVo5 OX 1689660 GN AEO54 301 PE 4 SV 1
tr A0A679DL48 A0A679DL48 9CAUD Tail completion protein OS Vibrio phage KIT04 OX 2508207 GN KIT04 151 PE 4 SV 1
tr A0A7S5R5K3 A0A7S5R5K3 9CAUD Tail completion protein OS Vibrio phage VPT02 OX 2698726 GN VPT02 154 PE 4 SV 1
tr A0A9E6MD33 A0A9E6MD33 9CAUD Tail completion protein OS Vibrio phage VPG01 OX 2798882 GN VPG01 154 PE 4 SV 1
tr A0A0B5H918 A0A0B5H918 9CAUD Tail completion protein OS Vibrio phage phi 3 OX 1589298 GN SBVP3 00136 PE 4 SV 1
tr A0A2D0YNK0 A0A2D0YNK0 9CAUD Tail completion protein OS Vibrio phage JSF10 OX 1983593 PE 4 SV 1
tr A0A5P8PM54 A0A5P8PM54 9CAUD Tail completion protein OS Serratia phage Slocum OX 2601679 GN CPT Slocum 178 PE 4 SV 1
tr A0A4Y5P1C1 A0A4Y5P1C1 9CAUD Putative tail completion protein OS Pantoea phage vB PagS AAS21 OX 2575261 GN AAS21 gp009 PE 4 SV 1
tr A0A873WTD3 A0A873WTD3 9CAUD Tail completion protein OS Providencia phage PSTCR5 OX 2783547 PE 4 SV 1
tr A0A2H4PRG8 A0A2H4PRG8 9CAUD Tail completion protein OS Proteus phage PM135 OX 2048008 PE 4 SV 1
tr J9QM89 J9QM89 9CAUD Tail completion protein OS Pectobacterium phage My1 OX 1204539 GN My1 139 PE 4 SV 1
tr A0A2D2W728 A0A2D2W728 9CAUD Tail completion protein OS Pectobacterium phage DU PP V OX 2041492 GN P13BB106kb p118 PE 4 SV 1
tr A0A5Q2U9R2 A0A5Q2U9R2 9CAUD Tail completion protein OS Bacteriophage Eos OX 2662137 PE 4 SV 1
tr A0A1I9SF31 A0A1I9SF31 9CAUD Tail completion protein OS Klebsiella phage vB Kpn IME260 OX 1912318 PE 4 SV 1
tr A0A5P8PKZ2 A0A5P8PKZ2 9CAUD Tail completion protein OS Klebsiella phage AmPh EK80 OX 2653643 GN AmPhEK80 0168 PE 4 SV 1
tr A0A5B8R6C9 A0A5B8R6C9 9CAUD Tail completion protein OS Klebsiella phage KpGranit OX 2596967 PE 4 SV 1
tr A0A5P8PLN4 A0A5P8PLN4 9CAUD Tail completion protein OS Klebsiella phage JIPh Kp127 OX 2653645 GN JIPhKp127 0161 PE 4 SV 1
tr A0A7R8MM18 A0A7R8MM18 9CAUD Tail completion protein OS Klebsiella phage vB KppS Anoxic OX 2762829 GN NHPMDKGM 00146 PE 4 SV 1
tr A0A7R8MM00 A0A7R8MM00 9CAUD Tail completion protein OS Klebsiella phage vB KaS Veronica OX 2762824 GN EKKBBHHG 00143 PE 4 SV 1
tr A0A5J6TB50 A0A5J6TB50 9CAUD Tail completion protein OS Salmonella phage vB SenS SB13 OX 2591135 PE 4 SV 1
tr A0A5B9N083 A0A5B9N083 9CAUD Tail completion protein OS Salmonella phage SE3 OX 2575327 PE 4 SV 1
tr A0A3P4A7P2 A0A3P4A7P2 9CAUD Tail completion protein OS Escherichia phage vB Eco mar003J3 OX 2419761 GN MAR003J3 00092 PE 4 SV 1
tr A0A5C0CER8 A0A5C0CER8 9CAUD Tail completion protein OS Salmonella phage SE19 OX 2592198 PE 4 SV 1
tr A0A7S9ST61 A0A7S9ST61 9CAUD Tail completion protein OS Salmonella phage GEC vB N7 OX 2777380 GN GECvBN7 gp176c PE 4 SV 1
tr A0A2K8HNC3 A0A2K8HNC3 9CAUD Tail completion protein OS Escherichia phage saus132 OX 2024325 GN P132 0142 PE 4 SV 1
tr A0A2Z5HTN5 A0A2Z5HTN5 9CAUD Tail completion protein OS Salmonella phage S124 OX 2231351 PE 4 SV 1
tr A0A6M3H7S2 A0A6M3H7S2 9CAUD Tail completion protein OS Salmonella phage vB SenS 3 OX 2713217 GN vBSenS3 193 PE 4 SV 1
tr A0A5J6T9E0 A0A5J6T9E0 9CAUD Tail completion protein OS Salmonella phage vB SenS SB13 OX 2591135 PE 4 SV 1
tr A0A5P1M866 A0A5P1M866 9CAUD Tail completion protein OS Escherichia phage vB EcoS 26175V OX 2576482 GN JOHFDMOO 00144 PE 4 SV 1
tr A0A5P1M906 A0A5P1M906 9CAUD Tail completion protein OS Escherichia phage vB EcoS 26175III OX 2576480 GN INCEGHDL 00163 PE 4 SV 1
tr I1TE48 I1TE48 9CAUD Tail completion protein OS Escherichia phage vB EcoS AKFV33 OX 2681605 GN orf148 PE 4 SV 1
tr A0A2Z5HN12 A0A2Z5HN12 9CAUD Tail completion protein OS Salmonella phage S131 OX 2231354 PE 4 SV 1
tr A0A2K8H7G1 A0A2K8H7G1 9CAUD Tail completion protein OS Bacteriophage T5 like pork29 OX 2024332 GN P29 0140 PE 4 SV 1
tr A0A193H118 A0A193H118 9CAUD Tail completion protein OS Shigella phage SHSML 45 OX 1863010 PE 4 SV 1
tr I7LH23 I7LH23 9CAUD Tail completion protein OS Yersinia phage phiR2 01 OX 1206557 GN g136 PE 4 SV 1
tr A0A161BZX7 A0A161BZX7 9CAUD Tail completion protein OS Salmonella phage NR01 OX 1647411 GN NR01 0077 PE 4 SV 1
tr A0A7L5CDP9 A0A7L5CDP9 9CAUD Tail completion protein OS Salmonella phage vB Sen I1 OX 2723910 GN vBSenI1 58 PE 4 SV 1
sp Q6QGE0 COMPL BPT5 Tail completion protein p143 OS Escherichia phage T5 OX 2695836 GN ORF136 PE 2 SV 1
tr A0A291NKM7 A0A291NKM7 9CAUD Tail completion protein OS Salmonella phage SP01 OX 1920294 PE 4 SV 1
p142
Tree scale: 1
tr A0A0B5H336 A0A0B5H336 9CAUD Tail completion protein OS Vibrio phage phi 3 OX 1589298 GN SBVP3 00135 PE 4 SV 1
tr A0A2D0Z3X5 A0A2D0Z3X5 9CAUD Tail completion protein OS Vibrio phage JSF10 OX 1983593 PE 4 SV 1
tr A0A0N9BAA6 A0A0N9BAA6 9CAUD Tail terminator protein OS Vibrio phage vB VorS PVo5 OX 1689660 GN AEO54 297 PE 4 SV 1
tr A0A679DNT0 A0A679DNT0 9CAUD Tail terminator protein OS Vibrio phage KIT04 OX 2508207 GN KIT04 150 PE 4 SV 1
tr A0A7S5QTD6 A0A7S5QTD6 9CAUD Tail terminator protein OS Vibrio phage VPT02 OX 2698726 GN VPT02 153 PE 4 SV 1
tr A0A9E6SMX0 A0A9E6SMX0 9CAUD Tail terminator protein OS Vibrio phage VPG01 OX 2798882 GN VPG01 153 PE 4 SV 1
tr A0A2H5BGC4 A0A2H5BGC4 9CAUD Tail completion protein OS Vibrio phage Ceto OX 2570300 GN CETO 28 PE 4 SV 1
tr A0A2H5BGY3 A0A2H5BGY3 9CAUD Tail completion protein OS Vibrio phage Thalassa OX 2570301 GN THALASSA 26 PE 4 SV 1
tr A0A6M9Z0Z3 A0A6M9Z0Z3 9CAUD Tail completion protein OS Vibrio phage AG74 OX 2736261 GN AG74 24 PE 4 SV 1
tr A0A7T8ENA9 A0A7T8ENA9 9CAUD Tail completion protein OS Vibrio phage GRLPWR OX 2801533 GN 28 PE 4 SV 1
tr A0A4Y6E7M4 A0A4Y6E7M4 9CAUD Tail completion protein OS Vibrio phage Brizo OX 2590896 GN 16 PE 4 SV 1
tr A0A6M9Z3C2 A0A6M9Z3C2 9CAUD Tail completion protein OS Vibrio phage Direpillow8 OX 2736264 GN DIREPILLOW8 29 PE 4 SV 1
tr A0A411BKQ6 A0A411BKQ6 9CAUD Tail tube terminator protein OS Vibrio phage VspSw 1 OX 2484249 GN VspSw1 153 PE 4 SV 1
tr A0A385E7Q6 A0A385E7Q6 9CAUD Tail tube terminator protein OS Vibrio phage vB VpS PG07 OX 2301664 PE 4 SV 1
tr A0A4Y5P1B5 A0A4Y5P1B5 9CAUD Tail terminator protein OS Pantoea phage vB PagS AAS21 OX 2575261 GN AAS21 gp010 PE 4 SV 1
tr A0A5P8PM26 A0A5P8PM26 9CAUD Tail tube terminator protein OS Serratia phage Slocum OX 2601679 GN CPT Slocum 177 PE 4 SV 1
tr A0A873WHS8 A0A873WHS8 9CAUD Tail terminator protein OS Providencia phage PSTCR5 OX 2783547 PE 4 SV 1
tr A0A2H4PRG0 A0A2H4PRG0 9CAUD Putative tail terminator protein OS Proteus phage PM135 OX 2048008 PE 4 SV 1
tr J9QNZ1 J9QNZ1 9CAUD Tail tube terminator protein OS Pectobacterium phage My1 OX 1204539 GN My1 138 PE 4 SV 1
tr A0A2D2W724 A0A2D2W724 9CAUD Tail completion protein OS Pectobacterium phage DU PP V OX 2041492 GN P13BB106kb p117 PE 4 SV 1
tr A0A5Q2UCN8 A0A5Q2UCN8 9CAUD Tail tube terminator protein OS Bacteriophage Eos OX 2662137 PE 4 SV 1
tr A0A1L6Z564 A0A1L6Z564 9CAUD Tail tube terminator protein OS Klebsiella phage vB Kpn IME260 OX 1912318 PE 4 SV 1
tr A0A5B8R3L2 A0A5B8R3L2 9CAUD Tail tube terminator protein OS Klebsiella phage KpGranit OX 2596967 PE 4 SV 1
tr A0A5P8PLE0 A0A5P8PLE0 9CAUD Tail tube terminator protein OS Klebsiella phage JIPh Kp127 OX 2653645 GN JIPhKp127 0160 PE 4 SV 1
tr A0A5P8PL06 A0A5P8PL06 9CAUD Tail tube terminator protein OS Klebsiella phage AmPh EK80 OX 2653643 GN AmPhEK80 0167 PE 4 SV 1
tr A0A7R8MM37 A0A7R8MM37 9CAUD Tail tube terminator protein OS Klebsiella phage vB KppS Anoxic OX 2762829 GN NHPMDKGM 00145 PE 4 SV 1
tr A0A7R8MLZ7 A0A7R8MLZ7 9CAUD Tail tube terminator protein OS Klebsiella phage vB KaS Veronica OX 2762824 GN EKKBBHHG 00142 PE 4 SV 1
tr I7J3V1 I7J3V1 9CAUD Tail tube terminator protein OS Yersinia phage phiR2 01 OX 1206557 GN g135 PE 4 SV 1
tr A0A2Z5HRT8 A0A2Z5HRT8 9CAUD Tail tube terminator protein OS Salmonella phage S124 OX 2231351 PE 4 SV 1
tr A0A5J6TAQ6 A0A5J6TAQ6 9CAUD Tail tube terminator protein OS Salmonella phage vB SenS SB13 OX 2591135 PE 4 SV 1
tr A0A5C0CEB3 A0A5C0CEB3 9CAUD Tail tube terminator protein OS Salmonella phage SE19 OX 2592198 PE 4 SV 1
tr A0A6M3H163 A0A6M3H163 9CAUD Tail tube terminator protein OS Salmonella phage vB SenS 3 OX 2713217 GN vBSenS3 192 PE 4 SV 1
tr A0A5P1M811 A0A5P1M811 9CAUD Tail tube terminator protein OS Escherichia phage vB EcoS 26175V OX 2576482 GN JOHFDMOO 00143 PE 4 SV 1
tr A0A5P1M952 A0A5P1M952 9CAUD Tail tube terminator protein OS Escherichia phage vB EcoS 26175III OX 2576480 GN INCEGHDL 00162 PE 4 SV 1
tr A0A7S9SSU5 A0A7S9SSU5 9CAUD Tail tube terminator protein OS Salmonella phage GEC vB N7 OX 2777380 GN GECvBN7 gp175c PE 4 SV 1
tr A0A3P4A7P4 A0A3P4A7P4 9CAUD Tail tube terminator protein OS Escherichia phage vB Eco mar003J3 OX 2419761 GN MAR003J3 00091 PE 4 SV 1
tr A0A2K8HTH8 A0A2K8HTH8 9CAUD Tail tube terminator protein OS Bacteriophage T5 like pork29 OX 2024332 GN P29 0139 PE 4 SV 1
tr A0A2Z5HQL8 A0A2Z5HQL8 9CAUD Tail tube terminator protein OS Salmonella phage S131 OX 2231354 PE 4 SV 1
tr A0A162EA65 A0A162EA65 9CAUD Tail tube terminator protein OS Salmonella phage NR01 OX 1647411 GN NR01 0076 PE 4 SV 1
tr I1TE47 I1TE47 9CAUD Tail tube terminator protein OS Escherichia phage vB EcoS AKFV33 OX 2681605 GN orf147 PE 4 SV 1
tr A0A193H1D5 A0A193H1D5 9CAUD Tail tube terminator protein OS Shigella phage SHSML 45 OX 1863010 PE 4 SV 1
tr A0A7L5CE47 A0A7L5CE47 9CAUD Tail tube terminator protein OS Salmonella phage vB Sen I1 OX 2723910 GN vBSenI1 57 PE 4 SV 1
tr A0A291NKM6 A0A291NKM6 9CAUD Tail tube terminator protein OS Salmonella phage SP01 OX 1920294 PE 4 SV 1
sp Q6QGE1 TTTP BPT5 Tail tube terminator protein p142 OS Escherichia phage T5 OX 2695836 GN ORF135 PE 1 SV 1

Bacteriophage TP901-1: TCP and THJP sequences

**TCP**	**ORF40**	
	MKSSLSFKGIDQLVKHLDKAASLKGVQQVVKSNTSNMTANMQKLVPVDTGYMKRSIKMEL<br>TEGGFSGQAGPHTDYSAYVEYGTRFQSAQPFVKPAYNEQKGVFIKDLERLLK	
**THJP**	**ORF41**	
	MIKTRDQSIFDELFKRIQALGYTVYDYKPMNEVGYPFVELENTQTIHEANKTDIKGTVSL<br>SLSVWGLQKKRKEVSDMASNIFNQALNISATDGYSWALNSQASTIQMLDDTTTNTPLKRA<br>LINLEFRLR	

blastp (version: BLASTP 2.12.0+)
Database: uniprotkb_refprotswissprot
Sequence: EMBOSS_001
Length: 112

Mon, Dec 11, 2023 at 10:17:08
Mon, Dec 11, 2023 at 10:17:33

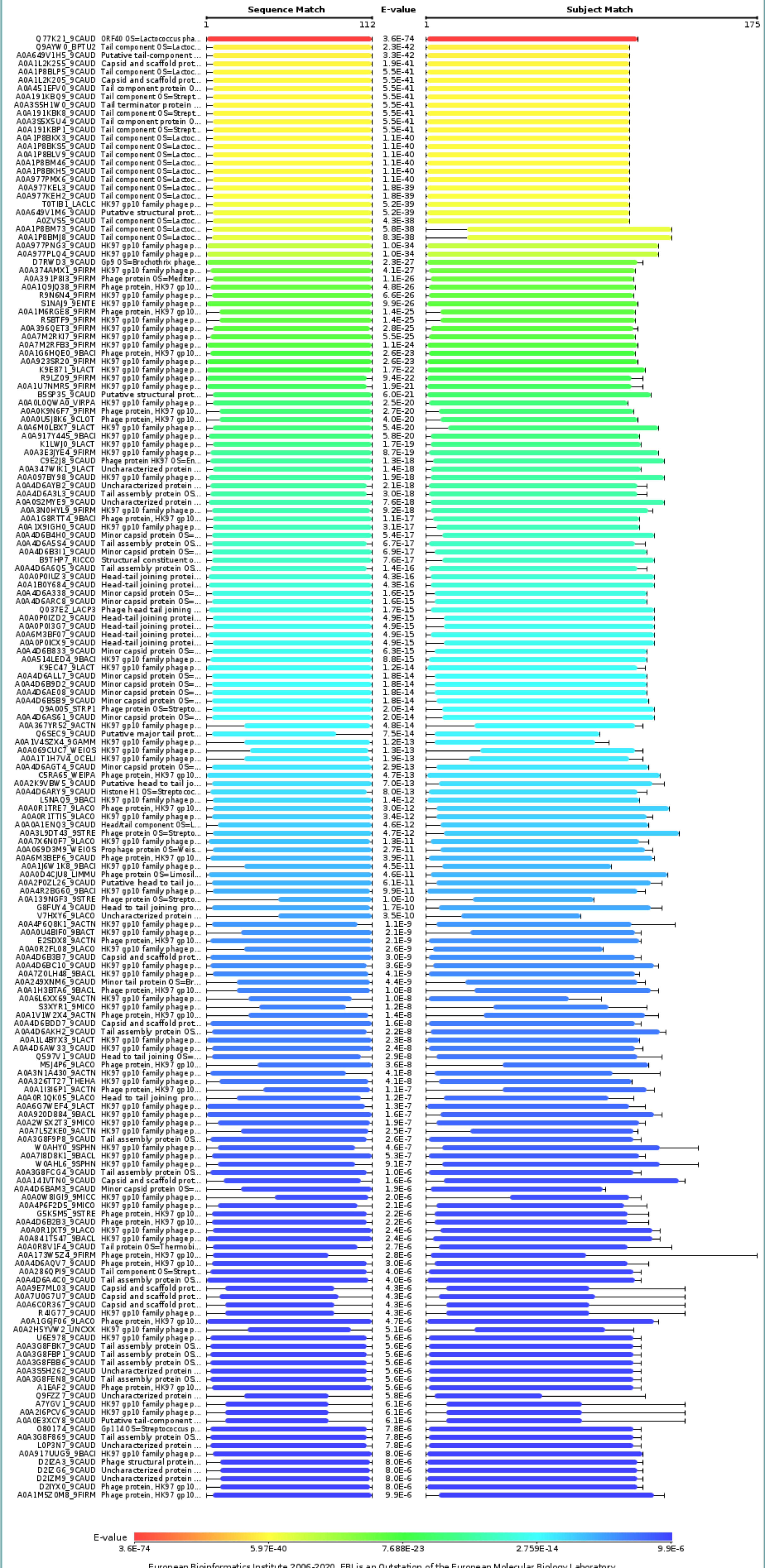

European Bioinformatics Institute 2006-2020. EBI is an Outstation of the European Molecular Biology Laboratory.

TP901-1

ORF40

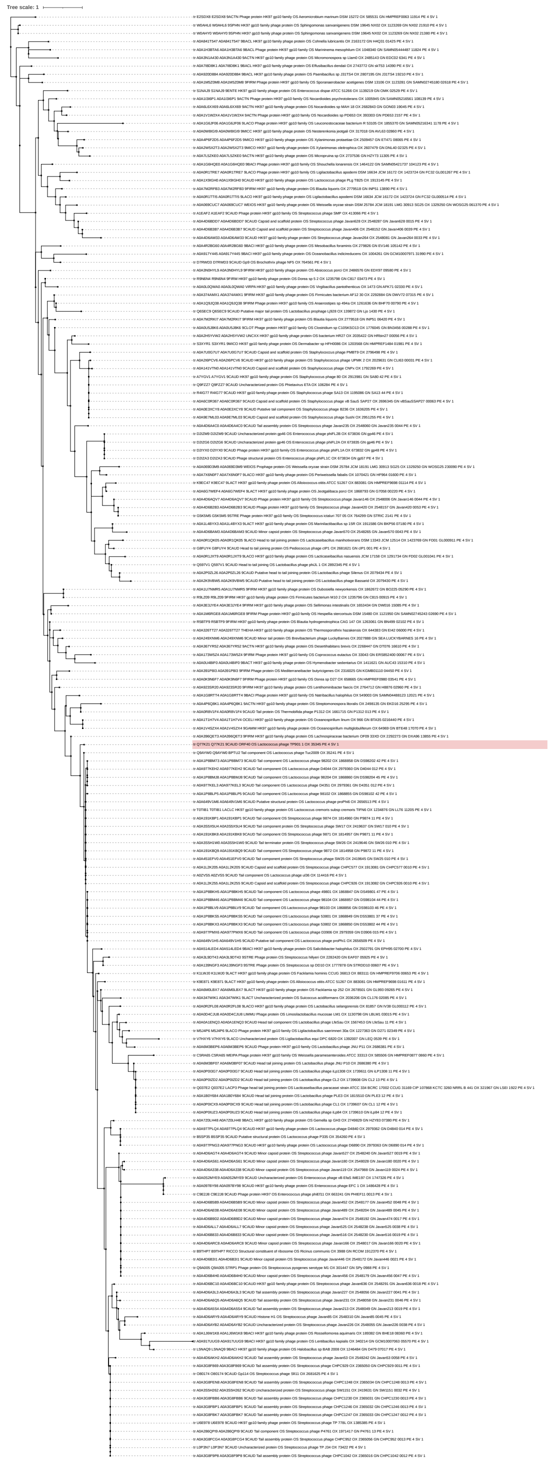

ORF41

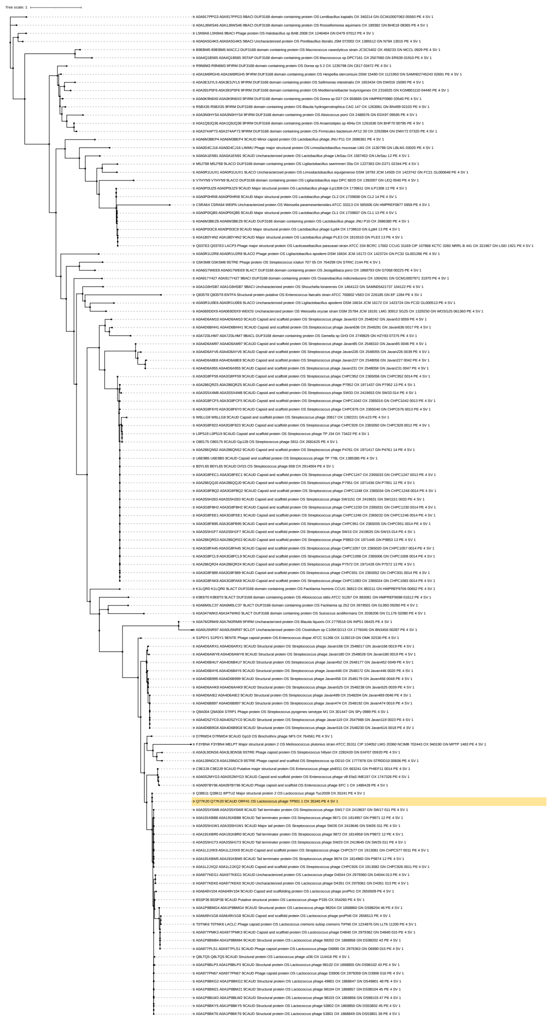

Bacteriophage HK97: TCP and THJP sequences

**TCP**	**gp10**
	MIETSLDFSGLNDIAKDLEALSRAENNKVLRDATRAGAEVLKEEVIDRAPVRTGKLKKNV<br>VVVTQKSRRRGEISSGVHIRGVNPRTGNSDNTMKANNPRNAFYWRFVELGTANMPAHPFV<br>RPAYDTREEEAASVAIARMNQAIDEVLSK
**THJP**	**gp11**
	MNEDNIYALLSPLAEGRVYPYVAPLGSDGKPSVSPPWIIFSIVDDVSADVLCGQAESRVS<br>VQVDVYSTSIAESRSLRDLVLASLEPLTPTEVVKIPGYEPDYRLYRATLDFKVTP

blastp (version: BLASTP 2.12.0+)
Database: uniprotkb_refprotswissprot
Sequence: EMBOSS_001
Length: 149

Mon, Dec 11, 2023 at 10:15:59
Mon, Dec 11, 2023 at 10:16:29

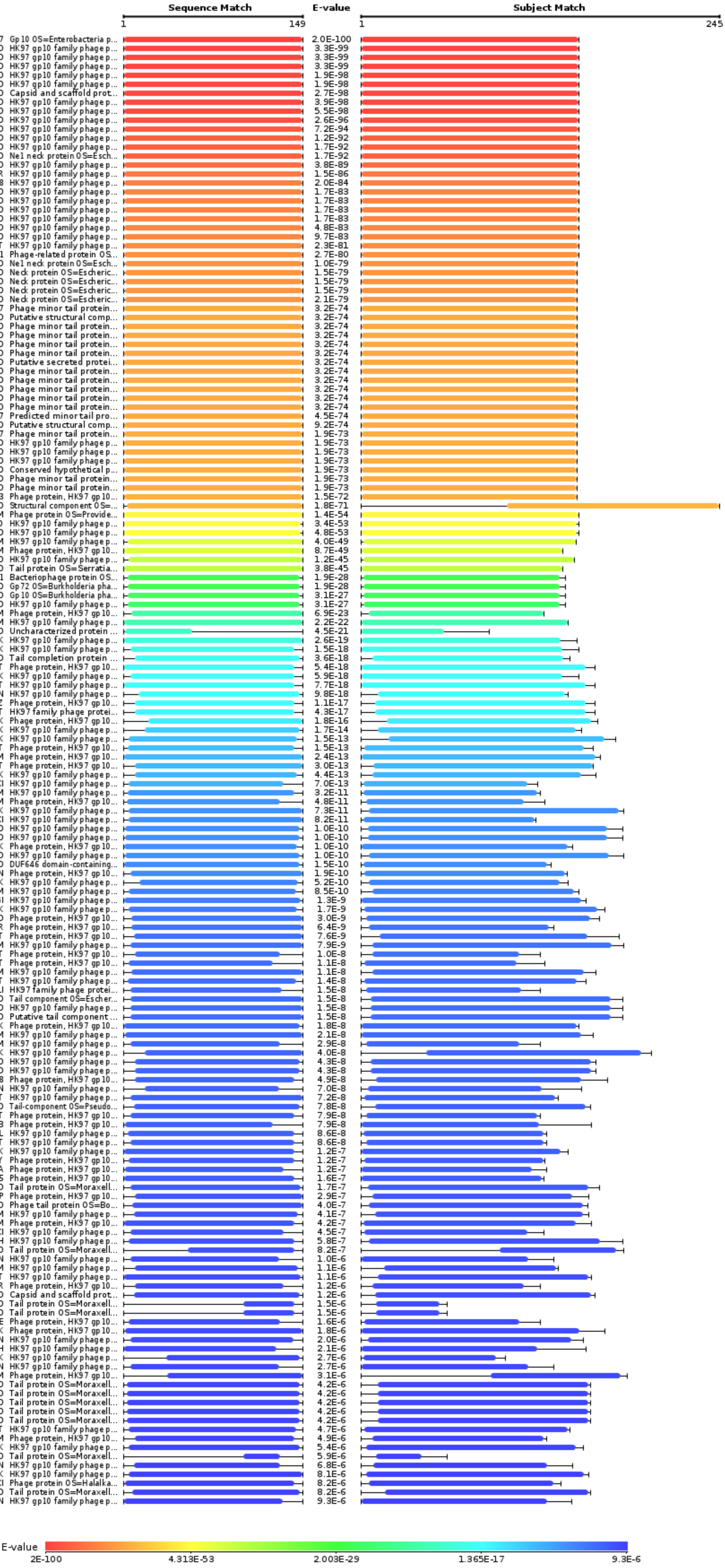

Sequence	Description	E-value
Q9MCS9_BPHK7	Gp10 OS=Enterobacteria p...	2.0E-100
K7P7Y7_9CAUD	HK97 gp10 family phage p...	3.3E-99
K7P6X4_9CAUD	HK97 gp10 family phage p...	3.3E-99
G8C7I9_9CAUD	HK97 gp10 family phage p...	3.3E-99
K7P724_9CAUD	HK97 gp10 family phage p...	1.9E-98
K7PH34_9CAUD	HK97 gp10 family phage p...	1.9E-98
A0A6B9WI65_9CAUD	Capsid and scaffold prot...	2.7E-98
K7PH04_9CAUD	HK97 gp10 family phage p...	3.9E-98
K7P712_9CAUD	HK97 gp10 family phage p...	5.5E-98
A0A2Z5H131_9CAUD	HK97 gp10 family phage p...	2.6E-96
A0A220NRP5_9CAUD	HK97 gp10 family phage p...	7.2E-94
K7P6I1_9CAUD	HK97 gp10 family phage p...	1.2E-92
K7PH84_9CAUD	HK97 gp10 family phage p...	1.7E-92
A0A653FUA5_9CAUD	Ne1 neck protein OS=Esch...	1.7E-92
F1C579_9CAUD	HK97 gp10 family phage p...	3.8E-89
A0A8J7GU45_9ENTR	HK97 gp10 family phage p...	1.5E-86
A7MLQ4_CROS8	HK97 gp10 family phage p...	2.0E-84
A0A482ME45_9CAUD	HK97 gp10 family phage p...	1.7E-83
A0A482J4N0_9CAUD	HK97 gp10 family phage p...	1.7E-83
A0A482J1T3_9CAUD	HK97 gp10 family phage p...	1.7E-83
A0A2Z2FCP9_9CAUD	HK97 gp10 family phage p...	1.7E-83
A0A482J438_9CAUD	HK97 gp10 family phage p...	4.8E-83
A0A482MGU6_9CAUD	HK97 gp10 family phage p...	9.7E-83
A0A4Z0L1A5_SALET	HK97 gp10 family phage p...	2.3E-81
A0A0H2YZV1_ECOK1	Phage-related protein OS...	2.7E-80
A0A653FVD6_9CAUD	Ne1 neck protein OS=Esch...	1.0E-79
A0A9E7E2F0_9CAUD	Neck protein OS=Escheric...	1.5E-79
A0A9E7J5W9_9CAUD	Neck protein OS=Escheric...	1.5E-79
A0A9E7E3U3_9CAUD	Neck protein OS=Escheric...	1.5E-79
A0A9E7E2J5_9CAUD	Neck protein OS=Escheric...	2.1E-79
Q8X5F4_ECO57	Phage minor tail protein...	3.2E-74
Q6H9U2_9CAUD	Putative structural comp...	3.2E-74
A0A7R7HL67_9CAUD	Phage minor tail protein...	3.2E-74
A0A7R7D2E4_9CAUD	Phage minor tail protein...	3.2E-74
A0A7R7HL81_9CAUD	Phage minor tail protein...	3.2E-74
A0A7R7HKX2_9CAUD	Phage minor tail protein...	3.2E-74
B6ETF2_9CAUD	Putative secreted protei...	3.2E-74
A0A7R7HKT5_9CAUD	Phage minor tail protein...	3.2E-74
A0A7R7D0J1_9CAUD	Phage minor tail protein...	3.2E-74
A0A7R7HLK4_9CAUD	Phage minor tail protein...	3.2E-74
A0A7R7D1K9_9CAUD	Phage minor tail protein...	3.2E-74
A0A7U3LQI4_9CAUD	Phage minor tail protein...	3.2E-74
B7UR45_ECO27	Predicted minor tail pro...	4.5E-74
H6WZL6_9CAUD	Putative structural comp...	9.2E-74
A0A0H3JFL7_ECO57	Phage minor tail protein...	1.9E-73
A0A0P0ZD51_9CAUD	HK97 gp10 family phage p...	1.9E-73
B6DZA4_9CAUD	HK97 gp10 family phage p...	1.9E-73
A0A7G3RZY8_9CAUD	HK97 gp10 family phage p...	1.9E-73
B6DZY0_9CAUD	Conserved hypothetical p...	1.9E-73
A0A7R7HL87_9CAUD	Phage minor tail protein...	1.9E-73
A0A7R7D0R5_9CAUD	Phage minor tail protein...	1.9E-73
B2TWN1_SHIB3	Phage protein, HK97 gp10...	1.5E-72
A0A9E7E302_9CAUD	Structural component OS=...	1.8E-71
A0A1B7K1I9_9GAMM	Phage protein OS=Provide...	1.4E-54
M1SBC6_MORMO	HK97 gp10 family phage p...	3.4E-53
A0A1W6JP15_9CAUD	HK97 gp10 family phage p...	4.8E-53
A0A3D9UFP5_9GAMM	HK97 gp10 family phage p...	4.0E-49
A0A083ZZM4_9GAMM	Phage protein, HK97 gp10...	8.7E-49
A0A1P8DTH7_9CAUD	HK97 gp10 family phage p...	1.2E-45
A0A7G9V405_9CAUD	Tail protein OS=Serratia...	3.8E-45
A0A0H3KMM0_BURM1	Bacteriophage protein OS...	1.9E-28
Q3HQT4_9CAUD	Gp72 OS=Burkholderia pha...	1.9E-28
Q8W6U1_9CAUD	Gp10 OS=Burkholderia pha...	3.1E-27
C7BGH3_9CAUD	HK97 gp10 family phage p...	3.1E-27
A0A0G3CHS6_9GAMM	Phage protein, HK97 gp10...	6.9E-23
A0A1B9N5U1_9GAMM	HK97 gp10 family phage p...	2.2E-22
A0A1B5FP88_9CAUD	Uncharacterized protein ...	4.5E-21
A0A6G8I748_9BURK	HK97 gp10 family phage p...	2.6E-19
A0A0Q5HCA2_9BURK	HK97 gp10 family phage p...	1.5E-18
A0A5B8RN28_9CAUD	Tail completion protein ...	3.6E-18
A0A1V3TTM6_9PAST	Phage protein, HK97 gp10...	5.4E-18
A0A0Q5H8D6_9BURK	HK97 gp10 family phage p...	5.9E-18
A0A011P4V7_9PAST	HK97 gp10 family phage p...	7.7E-18
A0A5D0X9F7_9SPHN	HK97 gp10 family phage p...	9.8E-18
A6VNQ6_ACTSZ	Phage protein, HK97 gp10...	1.1E-17
E6KYB4_9PAST	HK97 family phage protei...	4.3E-17
G2JAU4_9BURK	Phage protein, HK97 gp10...	1.8E-16
A0A936ZGC8_9BURK	HK97 gp10 family phage p...	1.7E-14
A0A315B4H6_9BURK	HK97 gp10 family phage p...	1.5E-13
A0A2M8RY41_9PAST	Phage protein, HK97 gp10...	1.5E-13
I8T8A8_9GAMM	Phage protein, HK97 gp10...	2.4E-13
A0A379CB64_9PAST	Phage protein, HK97 gp10...	3.0E-13
A0A927FJP5_9BURK	HK97 gp10 family phage p...	4.4E-13
A0A2N5GVG1_9BACI	HK97 gp10 family phage p...	7.0E-13
A0A6I0F2S9_9FIRM	HK97 gp10 family phage p...	3.2E-11
A0A1M5Z0M8_9FIRM	Phage protein, HK97 gp10...	4.8E-11
A0A126Z885_9BURK	HK97 gp10 family phage p...	7.3E-11
A0A0V8JS20_9BACI	HK97 gp10 family phage p...	8.2E-11
K7P6S5_9CAUD	HK97 gp10 family phage p...	1.0E-10
K7PGX7_9CAUD	HK97 gp10 family phage p...	1.0E-10
A0A1H2PS93_9BURK	Phage protein, HK97 gp10...	1.0E-10
K7PM60_9CAUD	HK97 gp10 family phage p...	1.0E-10
G3ENA5_9CAUD	DUF646 domain-containing...	1.5E-10
A0A5C5XP60_9PLAN	Phage protein, HK97 gp10...	1.9E-10
A0A1Y0EN56_9BURK	HK97 gp10 family phage p...	5.2E-10
A0A1S1NVY6_9GAMM	HK97 gp10 family phage p...	8.5E-10
N8YGI6_ACIGI	HK97 gp10 family phage p...	1.3E-9
A0A2D2DSE2_9BURK	HK97 gp10 family phage p...	1.7E-9
A0A1J0GUY2_9CAUD	Phage protein, HK97 gp10...	3.0E-9
F0SQQ9_RUBBR	Phage protein, HK97 gp10...	6.4E-9
A0A518GZL0_9BACT	Phage protein, HK97 gp10...	7.6E-9
A0A4R1NIK8_9GAMM	HK97 gp10 family phage p...	7.9E-9
A0A1T5K2F0_9CLOT	Phage protein, HK97 gp10...	1.0E-8
A0A161YSG2_9CLOT	Phage protein, HK97 gp10...	1.1E-8
A0A1S1P2K6_9GAMM	HK97 gp10 family phage p...	1.1E-8
A0A858RF84_9BACT	HK97 gp10 family phage p...	1.4E-8
A0A069RGG4_PEPLI	HK97 family phage protei...	1.5E-8
A0A9E7E5E4_9CAUD	Tail component OS=Escher...	1.5E-8
A0A286S249_9CAUD	HK97 gp10 family phage p...	1.5E-8
A0A482IGW8_9CAUD	Putative tail component ...	1.5E-8
A0A1I1DNB4_9BURK	Phage protein, HK97 gp10...	1.8E-8
A0A139SVR5_9GAMM	HK97 gp10 family phage p...	2.1E-8
A0A926EU17_9FIRM	HK97 gp10 family phage p...	2.9E-8
A0A857J8M9_9BURK	HK97 gp10 family phage p...	4.0E-8
A0A6M5CBY9_9CAUD	HK97 gp10 family phage p...	4.3E-8
A0A6M5CBX6_9CAUD	HK97 gp10 family phage p...	4.3E-8
B2JL16_PARP8	Phage protein, HK97 gp10...	4.9E-8
A0A7X6BHV7_9SPHN	HK97 gp10 family phage p...	7.0E-8
A0A1U7CNK3_9BACT	HK97 gp10 family phage p...	7.2E-8
A0A9E7C708_9CAUD	Tail-component OS=Pseudo...	7.8E-8
A0A2T0B3P7_9CLOT	Phage protein, HK97 gp10...	7.9E-8
A0A239Q2J4_9RHOB	Phage protein, HK97 gp10...	7.9E-8
A0A7W1XD58_9BACL	HK97 gp10 family phage p...	8.6E-8
A0A2Z4WAQ7_9CLOT	HK97 gp10 family phage p...	8.6E-8
A0A0S9NF99_9BURK	HK97 gp10 family phage p...	1.2E-7
W6N7H7_CLOTY	Phage protein, HK97 gp10...	1.2E-7
A0A0H3J694_CLOPA	Phage protein, HK97 gp10...	1.2E-7
F4A0G8_MAHA5	Phage protein, HK97 gp10...	1.6E-7
A0A0R6PHU8_9CAUD	Tail protein OS=Moraxell...	1.7E-7
B1Y110_LEPCP	Phage protein, HK97 gp10...	2.9E-7
A0A261URU1_9BORD	Phage tail protein OS=Bo...	4.0E-7
A0A1E8CG92_9GAMM	HK97 gp10 family phage p...	4.1E-7
A0A1H2RD01_9GAMM	Phage protein, HK97 gp10...	4.2E-7
A0A0K9GSF3_9BACI	HK97 gp10 family phage p...	4.5E-7
A0A7S8EIA2_9HYPH	HK97 gp10 family phage p...	5.8E-7
A0A0R6PFZ3_9CAUD	Tail protein OS=Moraxell...	8.2E-7
W1S0Q3_9SPHN	HK97 gp10 family phage p...	1.0E-6
A0A942QPN9_9FIRM	HK97 gp10 family phage p...	1.1E-6
A0A1Y4GD33_9BACT	HK97 gp10 family phage p...	1.1E-6
A0A1U7M5J8_TISCR	Phage protein, HK97 gp10...	1.2E-6
A0A9E7QNL6_9CAUD	Capsid and scaffold prot...	1.2E-6
A0A0R6PJR3_9CAUD	Tail protein OS=Moraxell...	1.5E-6
A0A0R6PK65_9CAUD	Tail protein OS=Moraxell...	1.5E-6
F4LTS3_TEPAE	Phage protein, HK97 gp10...	1.6E-6
A0A6J5DWB5_9BURK	Phage protein, HK97 gp10...	1.8E-6
A0A0N0M3G1_9SPHN	HK97 gp10 family phage p...	2.0E-6
A0A364JTJ6_9HYPH	HK97 gp10 family phage p...	2.1E-6
A0A6J5B1X7_9BURK	HK97 gp10 family phage p...	2.7E-6
A0A0J7XUI4_9SPHN	HK97 gp10 family phage p...	2.7E-6
A0A1N7G4F6_9GAMM	Phage protein, HK97 gp10...	3.1E-6
A0A0R6PGZ1_9CAUD	Tail protein OS=Moraxell...	4.2E-6
A0A0R6PI09_9CAUD	Tail protein OS=Moraxell...	4.2E-6
A0A0R6PDD9_9CAUD	Tail protein OS=Moraxell...	4.2E-6
A0A0R6PCA5_9CAUD	Tail protein OS=Moraxell...	4.2E-6
A0A0R6PGM8_9CAUD	Tail protein OS=Moraxell...	4.2E-6
A0A6M0RDL4_9CLOT	HK97 gp10 family phage p...	4.7E-6
A0A1M5PVR9_9FIRM	Phage protein, HK97 gp10...	4.9E-6
A0A0Q6W313_9BURK	HK97 gp10 family phage p...	5.4E-6
A0A0R6PE69_9CAUD	Tail protein OS=Moraxell...	5.9E-6
A0A0S9CJS7_9SPHN	HK97 gp10 family phage p...	6.8E-6
A0A0S9MWX5_9BURK	HK97 gp10 family phage p...	8.1E-6
W4Q503_9BACI	Phage protein OS=Halalka...	8.2E-6
A0A0R6PHF1_9CAUD	Tail protein OS=Moraxell...	8.2E-6
W0AHY0_9SPHN	HK97 gp10 family phage p...	9.3E-6

European Bioinformatics Institute 2006-2020. EBI is an Outstation of the European Molecular Biology Laboratory.

HK97

gp10

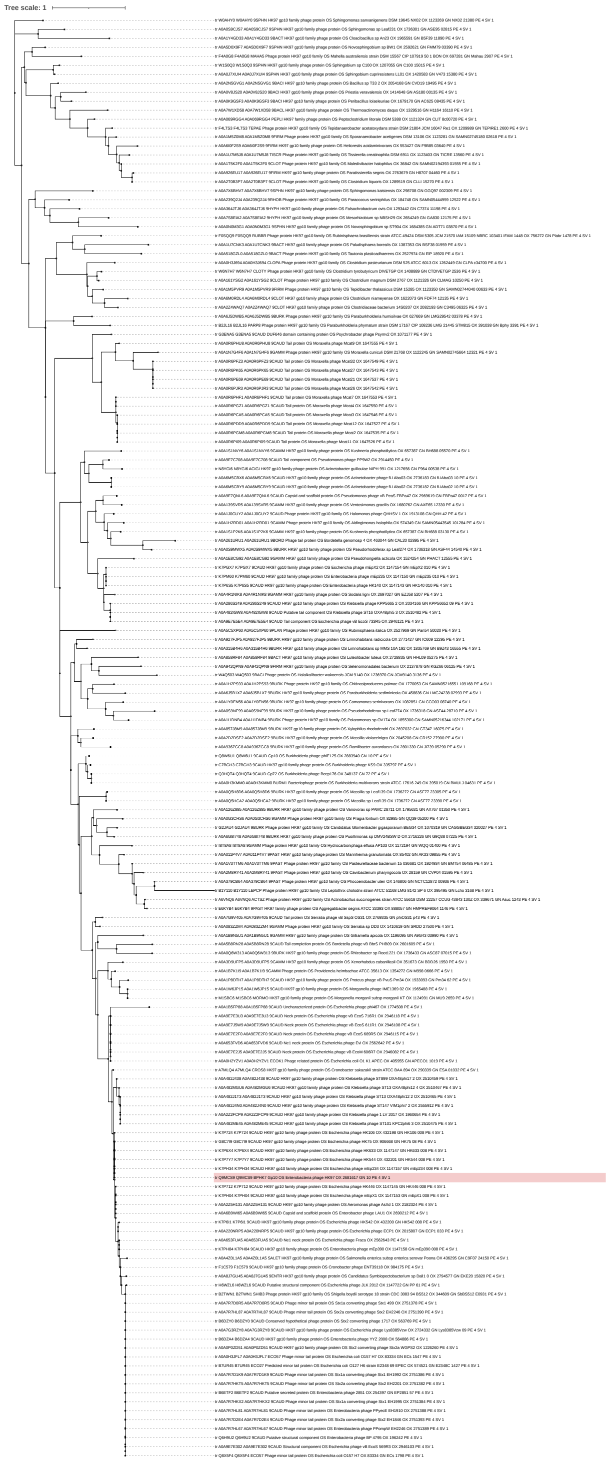

gp11

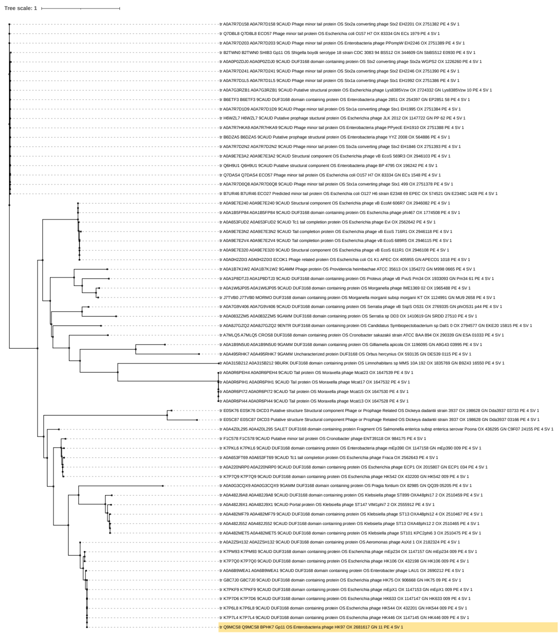

Bacteriophage P2: TCP and THJP sequences

**TCP**	**gpS**	
	MNEFKRFEDRLTGLIESLSPSGRRRLSAELAKRLRQSQQRRVMAQKAPDGTPYAPRQQQS<br>VRKKTGRVKRKMFAKLITSRFLHIRASPEQASMEFYGGKSPKIASVHQFGLSEENRKDGK<br>KIDYPARPLLGFTGEDVQMIEEIILAHLER	
**THJP**	**gpR**	
	MLKPDSLRRALTDAVTVLKTNPDMLRIFVDNGSIASTLAASLSFEKRYTLNVIVTDFTGD<br>FDLLIVPVLAWLRENQPDIMTTDEGQKKGFTFYADINNDSSFDISISLMLTERTLVSEVD<br>GALHVKNISEPPPPEPVTRPMELYINGELVSKWDE	

blastp (version: BLASTP 2.12.0+)
Database: uniprotkb_refprotswissprot
Sequence: EMBOSS_001
Length: 150

Mon, Dec 11, 2023 at 10:17:39
Mon, Dec 11, 2023 at 10:18:11

Sequence Match (1–150) · E-value · Subject Match (1–244)

Hit	Description	E-value
VPS_BPP2	Tail completion protein ...	1.8E-101
A0A653FSD8_9CAUD	Phage Nel neck protein O...	2.1E-100
A0A0H2YY71_ECOK1	Tail protein OS=Escheric...	2.1E-100
A0A7D3V1Y3_9CAUD	Phage virion morphogenes...	2.1E-100
A0A0F7LBV9_9CAUD	Tail completion protein ...	2.1E-100
A0A653FSD3_9CAUD	Phage Nel neck protein O...	6.2E-100
A0A7D3UWI5_9CAUD	Phage virion morphogenes...	6.2E-100
A0A653FS58_9CAUD	Phage Nel neck protein O...	1.8E-99
A0A0F7LDR6_9CAUD	Tail completion protein ...	1.8E-99
M1TAR9_9CAUD	Phage tail completion pr...	3.6E-99
A0A653FSM8_9CAUD	Phage Nel neck protein O...	5.1E-99
A0A9E7LPZ2_9CAUD	Virion morphogenesis pro...	1.0E-98
B7MEB5_ECO45	Tail completion protein ...	1.5E-98
A0A5P1M239_9CAUD	Phage virion morphogenes...	2.1E-98
A0A5P1M3D2_9CAUD	Phage virion morphogenes...	2.1E-98
A0A7D3QLV6_9CAUD	Phage virion morphogenes...	2.9E-98
A0A1L4BLZ5_9CAUD	Phage virion morphogenes...	5.9E-98
A0A5P1MBD9_9CAUD	Tail completion protein ...	4.2E-50
O80313_BP186	O protein OS=Escherichia...	6.3E-49
A0A9E7SQH8_9VIRU	Virion morphogenesis pro...	1.8E-48
A0A5C0CGX8_9CAUD	Virion morphogenesis pro...	2.6E-48
A0A7H0ENQ9_KLEVA	Phage virion morphogenes...	7.3E-48
A0A0M3UL83_9CAUD	Tail completion protein ...	3.0E-47
A0A378AHJ0_KLEPO	Phage tail completion pr...	6.0E-47
H2IRI4_RAHAC	Phage virion morphogenes...	6.5E-47
A0A376DMH0_9GAMM	Mu-like prophage protein...	8.4E-47
A0A1C4DMK0_9ENTR	Phage virion morphogenes...	1.2E-46
A0A482MG38_9CAUD	Virion morphogenesis pro...	2.4E-46
S4TP59_9CAUD	Phage virion morphogenes...	2.0E-45
A0A218M4K4_9CAUD	Tail completion protein ...	8.0E-45
A0A085GA08_9ENTR	Phage tail completion pr...	9.2E-44
A0A1I6E810_9ENTR	Phage virion morphogenes...	3.8E-43
A0A097QXF8_HAFAL	Tail protein OS=Hafnia a...	5.3E-43
A0A097R2L7_HAFAL	Tail protein OS=Hafnia a...	2.0E-40
A0A2N5EJ38_9GAMM	Phage virion morphogenes...	6.2E-39
A0A0U5L6M0_9GAMM	Tail protein OS=Duffyell...	6.1E-37
A0A328TQI5_9GAMM	Phage virion morphogenes...	8.2E-37
F1BUP7_9CAUD	Phage virion morphogenes...	1.4E-35
A0A0K6GU92_9NEIS	Phage virion morphogenes...	7.6E-35
A0A2U8I897_9GAMM	Phage virion morphogenes...	7.9E-35
A0A4R1NHS3_9GAMM	Phage virion morphogenes...	8.1E-35
A0A1S8CJS8_9GAMM	Phage virion morphogenes...	1.5E-34
A0A0Q4MP01_9GAMM	Phage tail protein OS=Er...	3.4E-34
A0A2U8I883_9GAMM	Phage virion morphogenes...	2.6E-33
A0A2I0CP34_9PSED	Phage virion morphogenes...	2.7E-33
E0WS42_9ENTR	Putative phage tail prot...	1.1E-32
A0A3M5NDF5_PSEAZ	Phage virion morphogenes...	3.1E-32
A0A0H3GWD6_KLEPH	Prophage, tail completio...	4.2E-32
A0A1V0B688_9PSED	Phage virion morphogenes...	1.8E-31
A0A3R8Z8L0_9GAMM	Phage virion morphogenes...	5.2E-31
R8ANB7_PLESH	Phage tail completion pr...	6.8E-31
A0A078LTA1_9PSED	Phage P2 essential tail ...	7.6E-31
A0A918NX88_9NEIS	Virion morphogenesis pro...	1.2E-30
A0A378YM38_9BURK	Mu-like prophage protein...	1.5E-30
S2KJZ0_HALAF	Tail protein OS=Halomona...	1.6E-30
Q5H265_XANOR	Phage-related tail prote...	2.7E-30
A0A1Y0KRX3_9PSED	Phage virion morphogenes...	3.2E-30
A0A010RQ35_PSEFL	Virion morphogenesis pro...	4.3E-30
A0A0J5P1S6_9PAST	Tail protein OS=Muribact...	6.6E-30
A0A0J5S1A7_9PAST	Tail protein OS=Muribact...	9.7E-30
A0A482J767_9CAUD	Virion morphogenesis pro...	1.5E-29
A0A482J928_9CAUD	Virion morphogenesis pro...	1.5E-29
A0A482IM24_9CAUD	Virion morphogenesis pro...	1.5E-29
A0A1N6Q2M3_9GAMM	Phage virion morphogenes...	2.9E-29
A0A2Z6GCU0_9PROT	Phage virion morphogenes...	3.5E-29
A0A0H3KK02_BURM1	Probable bacteriophage t...	4.5E-29
E5E3R3_9CAUD	Gp29 OS=Burkholderia pha...	4.5E-29
A0A427DM94_9PSED	Phage virion morphogenes...	5.1E-29
Q8P6I8_XANCP	Phage-related tail prote...	7.0E-29
C1DCF6_LARHH	Phage virion morphogenes...	8.3E-29
A0A0C5E288_9PSED	Phage virion morphogenes...	9.8E-29
B2FIY6_STRMK	Tail completion protein ...	1.0E-28
A0A941DMK5_9BURK	Phage virion morphogenes...	1.1E-28
A0A1H3DJ17_9PSED	Phage virion morphogenes...	1.1E-28
U3U522_9ENTR	Phage virion morphogenes...	1.4E-28
A0A1T0AVZ4_9PAST	Phage virion morphogenes...	1.4E-28
B2VHA9_ERWT9	Phage tail completion pr...	1.8E-28
A0A8D4LNE2_9PAST	Phage virion morphogenes...	2.0E-28
A0A1G7VM23_9GAMM	Phage virion morphogenes...	3.7E-28
A0A516SJJ7_9BURK	Phage virion morphogenes...	4.2E-28
Q9ZXL2_9CAUD	DNA, complete genome OS=...	4.4E-28
A0A3G8F195_9CAUD	Virion morphogenesis pro...	4.4E-28
A0A1M4Y3V5_9GAMM	Phage virion morphogenes...	5.3E-28
A4PE39_9CAUD	Tail completion protein ...	7.1E-28
Q8ZMU0_SALTY	Fels-2 prophage protein ...	1.0E-27
Q1QXR4_CHRSD	Phage virion morphogenes...	1.1E-27
V9IQH0_9CAUD	Tail completion protein ...	1.2E-27
A0A7I6GZJ7_ECOHS	Phage virion morphogenes...	2.0E-27
A0A873WD77_9CAUD	Tail completion protein ...	2.1E-27
S6HSB0_9GAMM	Phage virion morphogenes...	2.7E-27
A0A2Z2FG08_9CAUD	Tail completion protein ...	2.8E-27
A0A0H2V5M7_ECOL6	Putative phage tail prot...	4.0E-27
A0A140NDJ3_ECOBD	Phage virion morphogenes...	4.0E-27
A0A1S6L001_9CAUD	Tail completion protein ...	4.1E-27
A0A1S5NPT5_9CAUD	Putative virion morphoge...	4.1E-27
A0A0U4P1B7_9PSED	Phage tail protein OS=Ps...	4.3E-27
A0A1B2QYR4_9BORD	Phage tail protein OS=Bo...	5.2E-27
A0A4Z0PLH2_SALET	Phage virion morphogenes...	5.7E-27
A0A482J6S7_9CAUD	Virion morphogenesis pro...	5.7E-27
A0A077K9X5_9CAUD	Phage virion morphogenes...	8.1E-27
A0A4P8YFI6_9ENTR	Phage virion morphogenes...	1.1E-26
A0A7H0EQ75_KLEVA	Phage virion morphogenes...	2.3E-26
A0A8I0CTW3_9PSED	Phage virion morphogenes...	2.5E-26
A0A286D4T4_9GAMM	Phage virion morphogenes...	3.4E-26
A0A921TH38_9GAMM	Phage virion morphogenes...	3.6E-26
D8J079_HERSS	Bacteriophage tail compl...	3.8E-26
A0A1H3ZWD1_9GAMM	Phage virion morphogenes...	4.6E-26
A0A482MGD3_9CAUD	Virion morphogenesis pro...	4.6E-26
B7MJH5_ECO45	Phage tail completion pr...	7.2E-26
A0A0B2D2Y2_9PSED	Phage virion morphogenes...	9.2E-26
A0A2G3K3M9_9BURK	Phage virion morphogenes...	9.4E-26
E5G6N4_9CAUD	Tail completion protein ...	1.6E-25
A0A6I2IL22_9ENTR	Phage virion morphogenes...	3.6E-25
A0A1G9EQN3_9GAMM	Phage virion morphogenes...	3.9E-25
A0A3N9U3Z4_9GAMM	Phage virion morphogenes...	4.1E-25
A0A7X4FW67_9GAMM	Phage virion morphogenes...	5.2E-25
N2IMF4_9PSED	Phage virion morphogenes...	5.5E-25
A0A0C4Y807_9BURK	Phage tail completion pr...	7.9E-25
A0A7G1GMB5_9CAUD	Tail completion protein ...	8.2E-25
A0A482J5U1_9CAUD	Virion morphogenesis pro...	1.5E-24
A0A482J526_9CAUD	Virion morphogenesis pro...	1.5E-24
A0A482J6A5_9CAUD	Virion morphogenesis pro...	1.5E-24
A0A3S8ZPY3_9NEIS	Phage virion morphogenes...	1.5E-24
A0A1H7EQL8_9BURK	Phage virion morphogenes...	1.6E-24
W1IN05_9GAMM	Putative phage tail prot...	1.7E-24
A0A1E7PZU1_9GAMM	Phage virion morphogenes...	2.3E-24
A0A2S9GT28_9BURK	Phage virion morphogenes...	2.5E-24
A0A840RM05_9BURK	Phage virion morphogenes...	3.4E-24
A0A2C6DN00_9GAMM	Phage virion morphogenes...	3.5E-24
A0A3S0W483_9GAMM	Phage virion morphogenes...	4.6E-24
A0A1C3K396_9BURK	Phage tail completion pr...	5.1E-24
A0A3R9AGG2_9GAMM	Phage virion morphogenes...	5.3E-24
A0A1G8TIP3_9GAMM	Phage virion morphogenes...	5.9E-24
A0A1E7RCL8_9GAMM	Phage virion morphogenes...	6.2E-24
A0A2S4LWJ7_9BURK	Phage virion morphogenes...	7.9E-24
A0A1S1NXR5_9GAMM	Phage virion morphogenes...	9.0E-24
C1DBK5_LARHH	Phage virion morphogenes...	9.9E-24
D4GM45_PANAM	S OS=Pantoea ananatis (s...	1.2E-23
A0A0Q5LSR4_9BURK	Phage virion morphogenes...	1.5E-23
A0A1Z9Z2M3_9GAMM	Phage virion morphogenes...	1.7E-23
A0A516SHS4_9BURK	Phage virion morphogenes...	2.4E-23
E5FFH6_9CAUD	Gp23 OS=Burkholderia pha...	2.6E-23
A0A0H3GKL9_KLEPH	Phage virion morphogenes...	3.2E-23
A0A014MD39_9GAMM	Virion morphogenesis pro...	3.4E-23
B5JY62_9GAMM	Phage virion morphogenes...	3.9E-23
A0A923KMT7_9BURK	Phage virion morphogenes...	4.1E-23
A0A011P597_9PAST	Virion morphogenesis pro...	5.5E-23
D3VKL6_XENNA	Phage tail protein S OS=...	5.6E-23
I3DCI7_9PAST	Phage virion morphogenes...	5.6E-23
A0A4U1HST0_9BURK	Phage virion morphogenes...	8.9E-23
D8MP74_ERWBE	Phage tail protein OS=Er...	1.0E-22
A0A1V4B158_9PAST	Phage virion morphogenes...	1.0E-22
G0AIQ3_COLFT	Phage virion morphogenes...	1.4E-22
A0A235EW38_9RHOO	Phage virion morphogenes...	1.5E-22
A0A0M2WRG9_9BURK	Phage virion morphogenes...	1.6E-22
A0A559QNC3_9ALTE	Phage virion morphogenes...	1.7E-22
A0A944D816_DENS1	Phage virion morphogenes...	1.9E-22
A0A2T5J1G5_9GAMM	Phage virion morphogenes...	4.1E-22
A0A2M8RTA1_9PAST	Phage virion morphogenes...	4.3E-22
B8F4E8_GLAP5	Bacteriophage tail compl...	4.3E-22
A0A151UHS6_CAJCA	Tail completion protein ...	5.4E-22
A0A348HI73_9GAMM	Phage virion morphogenes...	5.6E-22
A0A1I7J4V0_9GAMM	Phage virion morphogenes...	5.9E-22
A0A432WB05_9GAMM	Phage virion morphogenes...	8.1E-22
A1VSG9_POLNA	Phage virion morphogenes...	9.0E-22
Q63LD4_BURPS	Bacteriophage tail compl...	9.0E-22
A0A4P1QFI7_9CAUD	Phage virion morphogenes...	9.0E-22
W7QGM5_9GAMM	Virion morphogenesis pro...	1.1E-21
A0A2A7UXR9_COMTR	Phage virion morphogenes...	1.1E-21
A0A4R1KIB6_9GAMM	Phage virion morphogenes...	1.7E-21
A0A4R3VAB8_9BURK	Phage virion morphogenes...	1.7E-21
E5E3V8_9CAUD	Gp29 OS=Burkholderia pha...	1.8E-21
A0A516SLV4_9BURK	Phage virion morphogenes...	2.5E-21
A0A876AEM0_9CAUD	P2 tail completion S-lik...	2.5E-21
A0A2N7UDK0_9GAMM	Phage virion morphogenes...	3.3E-21
Q63YM8_BURPS	Phage tail completion pr...	3.6E-21
A0A8F3ED29_9CAUD	Tail completion protein ...	3.6E-21
A0A2T6F191_9BURK	Phage virion morphogenes...	3.7E-21
A0A3N4VNG7_9GAMM	Phage virion morphogenes...	4.2E-21
A0A1E7WJE4_9BURK	Phage virion morphogenes...	5.4E-21
K4NZQ8_9CAUD	Phage tail completion pr...	6.8E-21
Q19UR2_9CAUD	Tail synthesis protein S...	9.3E-21
Q19UX3_9CAUD	Tail synthesis protein S...	9.3E-21
R9QBV1_9CAUD	Phage tail completion pr...	9.3E-21
A0A379C9X8_9PAST	Mu-like prophage protein...	1.9E-20
A0A840RI12_9NEIS	Phage virion morphogenes...	4.0E-20
A0A0D7K429_9BURK	Phage virion morphogenes...	4.5E-20
A0A514WLZ6_9SPHN	Phage virion morphogenes...	5.6E-20
A0A418WP26_9SPHN	Phage virion morphogenes...	6.2E-20
A0A7H0HG21_9BURK	Phage virion morphogenes...	7.8E-20
G6EFJ0_9SPHN	Phage virion morphogenes...	8.2E-20
R4JJX8_9CAUD	Phage virion morphogenes...	8.4E-20
A0A1W1XK61_9NEIS	Phage virion morphogenes...	1.5E-19
C5AGB5_BURGB	Phage virion morphogenes...	1.7E-19
A0A6N1X102_9BURK	Phage virion morphogenes...	2.2E-19
A0A157SJJ2_9BORD	Mu-like prophage protein...	3.1E-19
A0A077PFE8_XENBV	Putative phage tail prot...	4.1E-19
A0A519MN26_9BURK	Phage virion morphogenes...	4.4E-19
A0A363RFE7_9BURK	Phage virion morphogenes...	9.6E-19
A0A4P7L3D7_9GAMM	Phage virion morphogenes...	5.4E-18
A0A6N1X085_9BURK	Phage virion morphogenes...	1.4E-17
A0A4Q4AGX7_9BURK	Phage virion morphogenes...	1.6E-17
A0A3N1X8K1_9BURK	Phage virion morphogenes...	1.9E-17
C5A8Q9_BURGB	Phage virion morphogenes...	2.6E-17
R0MFQ0_NOSB1	Tail completion protein ...	1.4E-16
A0A8J7IP93_9SPHN	Phage virion morphogenes...	2.4E-16
A0A7W6RFV2_9PROT	Phage virion morphogenes...	1.2E-15
A0A7X4GD40_9SPHN	Phage virion morphogenes...	1.8E-15
G2H234_9ENTR	Phage virion morphogenes...	2.5E-15
A0A844Z4G1_9SPHN	Phage virion morphogenes...	5.3E-15
A0A1B8QD17_9GAMM	Phage virion morphogenes...	1.0E-14
C8NCA7_CARH6	Phage virion morphogenes...	1.3E-14
A0A2Z4UF55_9HYPH	Phage virion morphogenes...	6.2E-14
A0A1C3EE49_9GAMM	Phage virion morphogenes...	8.4E-14
A0A5M6I3S0_9PROT	Phage virion morphogenes...	1.5E-13
A0A5M6I3R0_9PROT	Phage virion morphogenes...	2.1E-13
A0A4V1LS68_9GAMM	Phage virion morphogenes...	2.7E-13
A0A7T8ACD0_9SPHN	Phage virion morphogenes...	2.8E-13
A0A118YKB1_9BURK	Phage tail protein OS=Bu...	5.1E-13
A0A1G7HWH3_9PROT	Phage virion morphogenes...	5.5E-13
A0A5D0XMG2_9SPHN	Phage virion morphogenes...	2.2E-12
J7SJI1_MORMO	Phage virion morphogenes...	4.1E-12
A0A1I2NX18_9SPHN	Phage virion morphogenes...	6.8E-12
A0A553ZS43_9BACI	Phage virion morphogenes...	6.2E-11
A0A1T0AS94_9PAST	Phage virion morphogenes...	6.3E-11
A0A143G7N8_9GAMM	Phage morphogenesis prot...	7.5E-11
A0A844Z718_9SPHN	Phage virion morphogenes...	1.0E-10
A0A381ECA4_9GAMM	Mu-like prophage protein...	1.6E-10
A0A437N1Y9_9SPHN	Phage virion morphogenes...	3.0E-10
A0A6N4T1Z2_9BURK	Phage virion morphogenes...	3.4E-10
N9WDU3_9SPHN	Phage virion morphogenes...	3.6E-10
G2JAP7_9BURK	Phage virion morphogenes...	4.4E-10
C8N771_CARH6	Phage virion morphogenes...	9.4E-10
A0A845A3G4_9SPHN	Phage virion morphogenes...	2.1E-9
F1Z9A8_9SPHN	Phage virion morphogenes...	3.1E-9
A0A918PE54_9SPHN	Phage virion morphogenes...	4.1E-9
A0A7W6CGM9_9SPHN	Phage virion morphogenes...	6.1E-9
A0A143DH82_9PROT	Phage virion morphogenes...	6.8E-9
A0A8J7H909_9ENTR	Phage virion morphogenes...	1.0E-8
A0A8J7KTY7_9ENTR	Phage virion morphogenes...	2.7E-8
A0A6G8AI73_9BURK	Phage virion morphogenes...	3.6E-8
A0A6I4T1N2_9SPHN	Phage virion morphogenes...	5.0E-8
T0H074_9SPHN	Virion morphogenesis pro...	5.4E-8
M1RL52_MORMO	Phage virion morphogenes...	1.3E-7
A0A5E7YM78_9SPHN	Phage tail completion pr...	1.7E-7
A0A2K2G613_9SPHN	Phage virion morphogenes...	2.8E-7
A0A8J7IGH9_9SPHN	Phage virion morphogenes...	4.0E-7
H2IY45_RAHAC	Phage virion morphogenes...	6.3E-7
A0A198GSD1_9GAMM	Tail completion protein ...	6.4E-7
A0A1I6EGS6_9ENTR	Phage virion morphogenes...	7.3E-7

E-value: 1.8E-101 · 3.625E-54 · 1.627E-30 · 1.09E-18 · 7.3E-7

European Bioinformatics Institute 2006-2020. EBI is an Outstation of the European Molecular Biology Laboratory.

P2

gpS

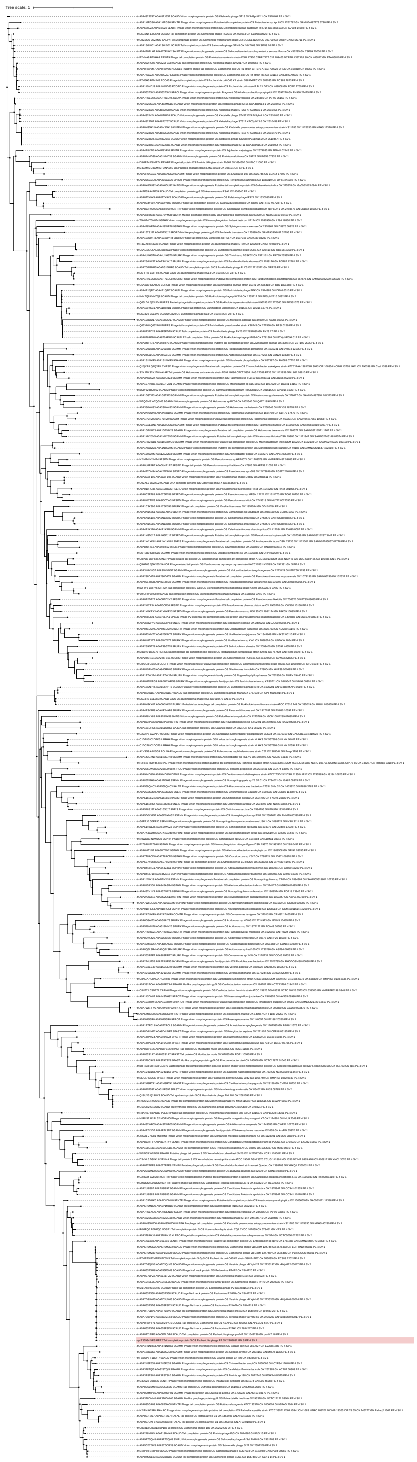

gpR

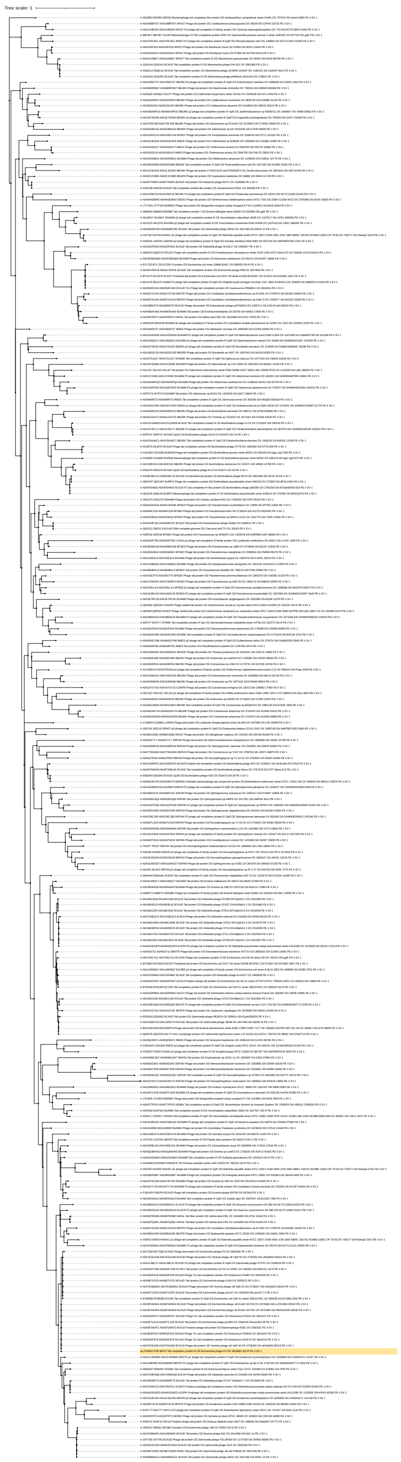

Bacteriophage Mu: TCP and THJP sequences

TCP **gpG**

MSLDMNVAVDVRRIQLALDELGTVTRDRAIPRVMAAALLSSTEQAFERQADPDTGKGWEA
WSDSWLAWRQDHGFVPGSILTLHGDLARSITTDYGQDYALIGSPKIYAAIHQWGGTPDMA
PRPAGVPARPYMGLDKTGEQEIFDAIRKRVSAALRQ

THJP **gpK**

MLEETEAALLARVRELFGATLRQVEPLTGTWTNEDVHRLFLAPPSVFLAWMGCGEGRTRR
EVESRWAFFVVAELLNGEPVNRPGIYQIVERLIAGVNGQTFGPTTGMRLTQVRNLCDDNR
INAGVVLYGVLFSGTTPLPSVVDLDSLDDYERHWQTWKFPDETPEFAAHINVNQEKDHDA
EN

blastp (version: BLASTP 2.12.0+)
Database: uniprotkb_refprotswissprot
Sequence: EMBOSS_001
Length: 156

Mon, Dec 11, 2023 at 10:15:43
Mon, Dec 11, 2023 at 10:16:12

Sequence Match: 1 – 156 | Subject Match: 1 – 207

Hit	Description	E-value
GPG_BPMU	Putative capsid assembly...	1.4E-110
C9DGN8_BPD10	Virion morphogenesis pro...	5.8E-110
A0A248KIU3_9ENTR	Phage virion morphogenes...	1.3E-62
A0A261QRZ7_9GAMM	Phage virion morphogenes...	3.0E-48
A0A0H3JJA5_ECO57	Phage morphogenesis prot...	4.1E-48
A0A8J7KWW5_9ENTR	Phage virion morphogenes...	1.2E-47
U3U0W8_9ENTR	Phage virion morphogenes...	3.2E-45
A0A081NL52_9GAMM	Phage virion morphogenes...	9.6E-31
D3V979_XENNA	Virion morphogenesis pro...	3.8E-28
W1IPH5_9GAMM	Putative virion morphoge...	3.1E-27
A0A0M2KFI4_9GAMM	Phage virion morphogenes...	1.9E-25
Q8EJ06_SHEON	Mu phage tail completion...	2.7E-24
W7QX48_9ALTE	Phage virion morphogenes...	3.9E-24
A0A2P9JZJ5_9CAUD	Virion morphogenesis pro...	1.1E-21
A0A2X4U7K5_9GAMM	Mu-like prophage protein...	6.8E-20
Q602Y2_METCA	Prophage MuMc02, virion ...	2.2E-18
A0A0F4QJZ2_9GAMM	Phage morphogeneis prote...	4.3E-17
A0A7G1H297_9BACT	Mu-like prophage FluMu G...	5.4E-17
M5DM48_9GAMM	Phage virion morphogenes...	7.3E-17
Q727Z6_DESVH	Virion morphogenesis pro...	7.8E-17
A0A3R8LCA8_BIBTR	Phage virion morphogenes...	4.0E-16
A0A1V4B3Z9_9PAST	Mu-like phage G protein ...	2.2E-15
A0A554WZN1_9BURK	Phage virion morphogenes...	5.2E-15
A0A2K9K025_9GAMM	Phage virion morphogenes...	7.4E-15
A0A2H6APP4_UNCXX	Phage virion morphogenes...	1.3E-14
A0A0C5DXT0_9PSED	Phage virion morphogenes...	2.4E-14
A0A239AKF7_9BACT	Phage virion morphogenes...	1.4E-13
A0A1H9FRV5_9GAMM	Mu-like prophage protein...	1.5E-13
VPG1_HAEIN	Mu-like prophage FluMu G...	2.0E-13
A0A0C1H503_9NEIS	Phage virion morphogenei...	2.0E-13
A0A376BT39_9NEIS	Mu-like prophage protein...	2.8E-13
A0A7W6RF58_9PROT	Phage virion morphogenes...	3.3E-13
Q7VPF5_HAEDU	Mu-like phage G protein ...	5.6E-13
G1UQM1_9BACT	Phage virion morphogenes...	1.8E-12
F5S494_KINKI	Prophage MuMc02 protein ...	2.0E-12
A0A2P1PSW5_9GAMM	Phage virion morphogenes...	3.7E-12
L7P7T2_9CAUD	Phage virion morphogenes...	5.1E-12
A0A125RNH6_9CAUD	Capsid and scaffold prot...	5.1E-12
A0A1T0B7W4_9PAST	Phage virion morphogenes...	5.9E-12
F2BF06_9NEIS	Prophage MuMc02 protein ...	6.1E-12
U3TXI2_9ENTR	Phage virion morphogenes...	6.5E-12
B8GL15_THISH	Phage virion morphogenes...	6.6E-12
A0A380N1M0_9GAMM	Mu-like prophage protein...	1.1E-11
A0A125RNC2_9CAUD	Capsid and scaffold prot...	1.4E-11
L7P7Z9_9CAUD	Phage virion morphogenes...	1.4E-11
A0A239L0D4_9PSED	Phage virion morphogenes...	1.6E-11
A0A2T5VCF6_9HYPH	Phage virion morphogenes...	1.6E-11
L7P833_9CAUD	Phage virion morphogenes...	2.0E-11
A0A3M5MMY2_PSEAZ	Phage virion morphogenes...	3.0E-11
A0A8J7KN65_9ENTR	Phage virion morphogenes...	3.5E-11
F5S6D6_KINKI	Prophage MuMc02 protein ...	4.7E-11
A0A381E401_9GAMM	Mu-like prophage protein...	5.2E-11
A0A1T2L982_9GAMM	Phage virion morphogenes...	5.3E-11
A0A7K3NMI0_9BACT	Phage virion morphogenes...	5.6E-11
L1P279_9NEIS	Phage virion morphogenei...	6.5E-11
A0A5P3MVL8_NEIAN	Phage virion morphogenes...	9.0E-11
A0A238TDR1_9NEIS	Phage virion morphogenes...	9.2E-11
A0A448VJU8_9NEIS	Phage-like protein OS=Ne...	9.2E-11
A0A8K1ZZ85_9CYAN	Phage virion morphogenes...	1.0E-10
A0A7W6WM60_9PROT	Phage virion morphogenes...	1.3E-10
A0A6L9LZG6_9BACT	Phage virion morphogenes...	1.6E-10
A0A0F3GUS2_9BACT	Phage virion morphogenes...	1.6E-10
H8GKL0_METAL	Phage virion morphogenes...	1.8E-10
A0A847SC97_9NEIS	Phage virion morphogenes...	2.1E-10
A7INX5_XANP2	Phage virion morphogenes...	2.3E-10
B7SDU0_9CAUD	Virion morphogenesis pro...	3.0E-10
A0A346FB18_9CAUD	Virion morphogenesis pro...	3.0E-10
A0A5Q3RZ35_9NEIS	Phage virion morphogenes...	3.5E-10
Q6TM73_BPD31	Virion morphogenesis pro...	4.2E-10
A0A3G8F3D3_9CAUD	Virion morphogenesis pro...	4.2E-10
L1P2M0_9NEIS	Phage virion morphogenei...	4.7E-10
A0A239BE06_9BACT	Phage virion morphogenes...	5.4E-10
A0A346FB73_9CAUD	Virion morphogenesis pro...	5.8E-10
A0A0S2SYG0_9CAUD	Virion morphogenesis pro...	5.8E-10
A0A220S3D8_9NEIS	Phage virion morphogenes...	6.8E-10
A0A238TDK5_9NEIS	Phage virion morphogenes...	1.3E-9
H8GJA7_METAL	Phage virion morphogenes...	1.3E-9
V7I9Z3_EIKCO	Phage virion morphogenes...	1.3E-9
D5APT0_RHOCB	Phage virion morphogenes...	1.5E-9
A0A096AIF1_9BURK	Phage morphogeneis prote...	1.6E-9
A0A0B5CHY5_NEIEG	Phage virion morphogenes...	2.5E-9
A0A1G9AWM7_9GAMM	Phage virion morphogenes...	3.1E-9
F3YW17_DESAF	Phage virion morphogenes...	3.1E-9
A0A972G8G6_9RHOB	Phage virion morphogenes...	5.3E-9
A0A1N6NVB0_9HYPH	Phage virion morphogenes...	6.6E-9
A0A8K1ZWL0_9CYAN	Phage virion morphogenes...	1.0E-8
A0A840FXM8_RHOTE	Phage virion morphogenes...	1.5E-8
F5RBW6_METUF	Phage virion morphogenes...	1.7E-8
A0A376D941_9GAMM	Mu-like prophage protein...	1.9E-8
A0A554XFW7_9BURK	Phage virion morphogenes...	2.2E-8
I0AP42_IGNAJ	Mu-like prophage protein...	2.2E-8
A0A7X1ZE46_9PROT	Phage virion morphogenes...	2.2E-8
Q7VLA6_HAEDU	Bacteriophage Mu G-like ...	2.7E-8
A0A6L9LZH1_9BACT	Phage virion morphogenes...	3.5E-8
C8NAX4_CARH6	Phage virion morphogenes...	4.2E-8
R9U1C4_9CAUD	Virion morphogenesis pro...	4.5E-8
A0A5P3MPP1_NEIAN	Phage virion morphogenes...	5.3E-8
A0A8J7V2L4_9PROT	Phage virion morphogenes...	5.8E-8
A0A6H0T9F8_9PAST	Phage virion morphogenes...	6.2E-8
A0A157SW55_9BORD	Mu-like prophage protein...	6.3E-8
A0A376DGU8_9GAMM	Mu-like prophage protein...	7.2E-8
A0A1V0PXS1_9RHOB	Phage capsid and scaffol...	7.8E-8
A0A7L9WM09_9RHOB	Phage virion morphogenes...	7.9E-8
A0A2D1GNX5_9CAUD	Virion morphogenesis pro...	8.4E-8
A0A0M3VI85_9CAUD	Virion morphogenesis pro...	1.7E-7
Q72D17_DESVH	Virion morphogenesis pro...	2.1E-7
I0AFP9_IGNAJ	Mu-like prophage protein...	2.2E-7
A0A0J5QI12_9RHOB	Phage virion morphogenes...	2.3E-7
Q0EWD6_9PROT	Possible bacteriophage M...	2.5E-7
A0A1N7Q1K4_9RHOB	Phage virion morphogenes...	2.8E-7
A0A1C2DEE2_9HYPH	Phage virion morphogenes...	2.9E-7
A0A0F7K9H5_9PROT	Phage virion morphogenes...	2.9E-7
A0A554XAZ5_9BURK	Phage virion morphogenes...	3.1E-7
A0A6P1UAL3_9GAMM	Phage virion morphogenes...	3.2E-7
A0A1I3FPC7_9RHOB	Phage virion morphogenes...	4.1E-7
A0A1V3RSI1_9BURK	Phage virion morphogenes...	7.1E-7
A0A0M2KAY7_9GAMM	Phage morphogeneis prote...	8.7E-7
A0A1W6CZ02_9RHOB	Phage virion morphogenes...	9.0E-7
A0A2R8BD47_9RHOB	Phage virion morphogenes...	1.3E-6
VPG2_HAEIN	Mu-like prophage FluMu G...	1.3E-6
A0A0F4NK93_9VIBR	Phage morphogeneis prote...	1.4E-6
A0A7Z2NVY0_9SPHN	Phage virion morphogenes...	1.6E-6
A0A1I4SUB9_9BACT	Phage virion morphogenes...	1.9E-6
A0A318T483_9HYPH	Phage virion morphogenes...	2.4E-6
B7SDN8_9CAUD	Mu-like prophage protein...	2.6E-6
A0A1X7P9W7_9RHOB	Phage virion morphogenes...	2.6E-6
A0A4R3NNR8_9HYPH	Phage virion morphogenes...	3.0E-6
A0A4R3NKJ6_9HYPH	Phage virion morphogenes...	3.0E-6
A0A833IQR5_9GAMM	Phage virion morphogenes...	3.2E-6
A0A6P1YPY5_9HYPH	Phage virion morphogenes...	3.3E-6
A0A0F7KAK8_9PROT	Phage virion morphogenes...	4.1E-6
A0A0Q3BFP2_9RHOB	Phage virion morphogenes...	4.5E-6
A0A2S5JEN2_9RHOB	Phage virion morphogenes...	5.0E-6
A0A5S9P390_9GAMM	Phage virion morphogenes...	5.5E-6
A0A1I5W3P0_9RHOB	Phage virion morphogenes...	5.8E-6
G4CJJ8_9NEIS	Prophage MuMc02 protein ...	5.9E-6
A0A378B1X3_KLEPO	Mu-like prophage protein...	5.9E-6
A0A3S1BVL1_ANAVA	Virion morphogenesis pro...	6.1E-6
A0A3N0V772_9GAMM	Phage virion morphogenes...	6.5E-6
Q6QIB8_BPBMU	Gp31 OS=Burkholderia pha...	6.5E-6
A0A2Z4PZG0_9CAUD	Virion morphogenesis pro...	6.6E-6
A0A4R5W2J0_9BURK	Phage virion morphogenes...	6.9E-6
A0A0Q3BIL0_9RHOB	Phage virion morphogenes...	7.0E-6
A0A547PW90_9RHOB	Phage virion morphogenes...	8.1E-6
A0A2I6S9F1_9RHOO	Phage virion morphogenes...	9.8E-6

E-value: 1.4E-110 — 3.704E-58 — 6.025E-32 — 7.684E-19 — 9.8E-6

European Bioinformatics Institute 2006-2020. EBI is an Outstation of the European Molecular Biology Laboratory.

Mu

gpG

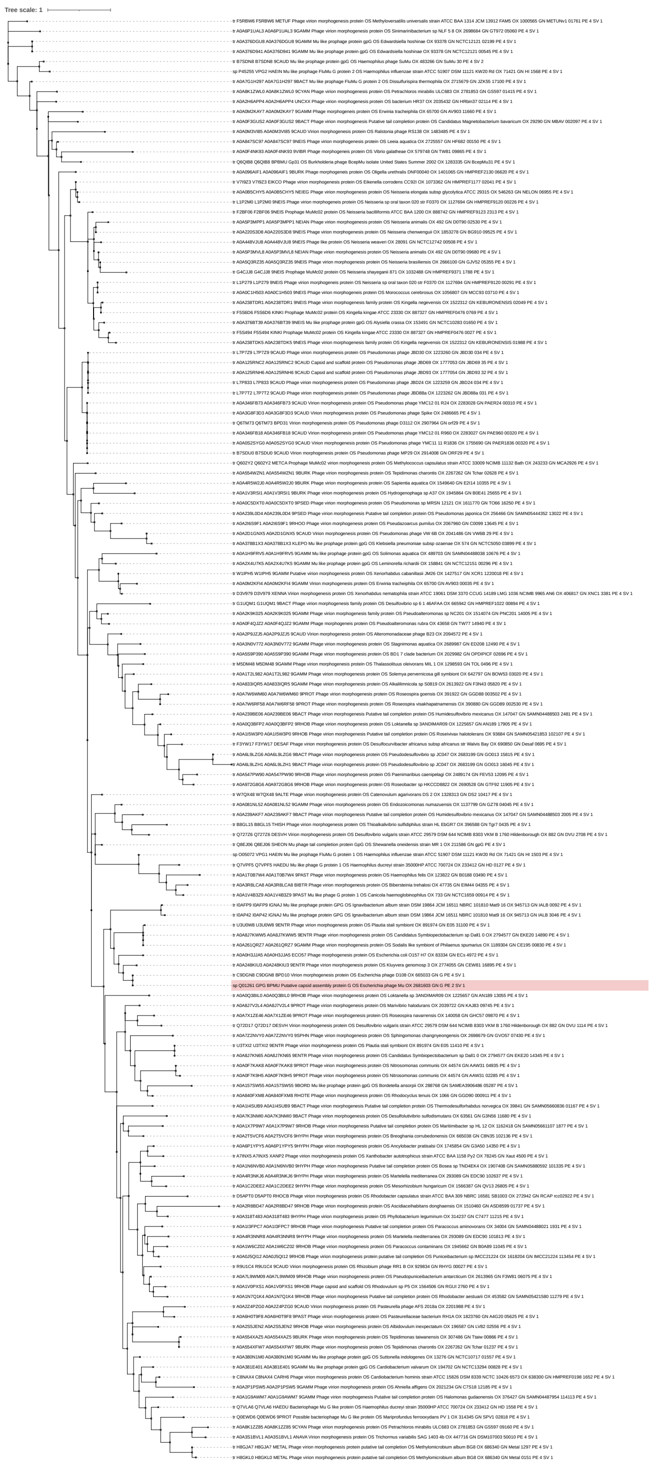

gpK

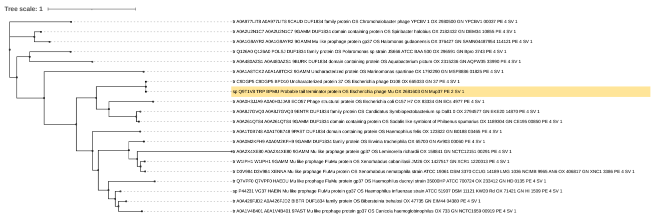

Supplementary Figure 6 | Homology search of TCPs with known function and of THJPs from the same phages. The analysis for each individual phage is presented in three successive pages.
The first page shows sequences of the TCP and of the THJP.
The second page shows the Protein Blast output of the homology search in the UniProtKB/Swiss-Prot non-redundant protein sequence database. The E-threshold was set to 0.00001. E-value scores are shown in the figure center. They are also displayed in the figure left and right using the color code shown in the figure bottom. The region on homology is presented for the query sequence (left) and for the target homologous protein identified (right). Note that the protein length scale is different in the left and right panels.
The third page shows a Newick display of the phylogeny of homologous TCPs (left) and THJP (right). Datasets of homologous proteins were generated with Protein Blast as above and their phylogeny was computed using the NGphylogeny automatic workflow using default parameters. Query TCP and THJP sequences are highlighted in salmon and yellow, respectively.

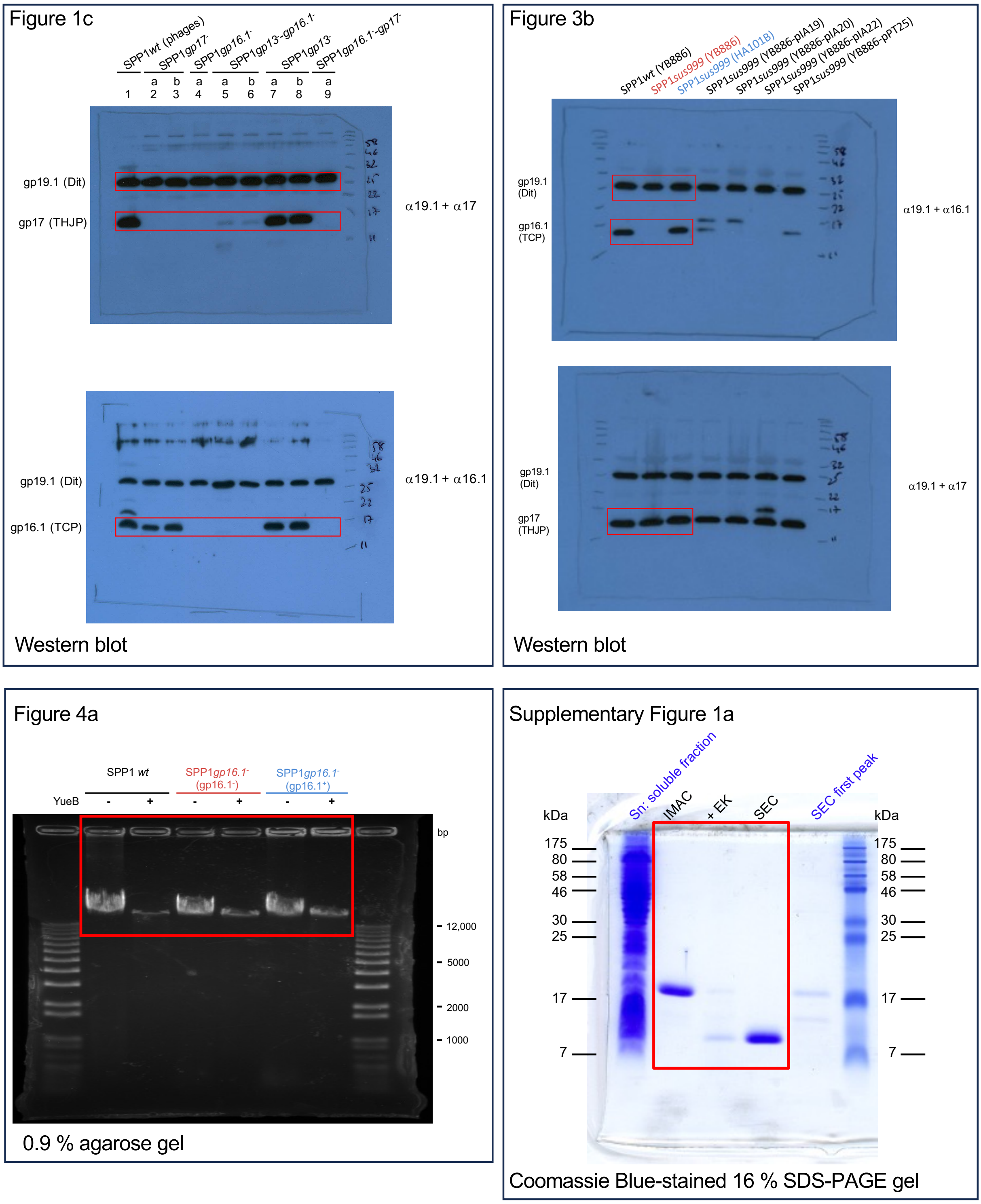

Supplementary Figure 7 | Uncropped and unedited images of X-ray films and gels shown in the main and supplementary figures. Western blot size markers were manually transferred onto X-ray films from colored markers present on the nitrocellulose transfer membranes. The red rectangles correspond to the parts of the images used in the main and supplementary figures indicated at the top left of each black rectangle.

Supplementary references

1. Hanahan, D. Studies on transformation of Escherichia coli with plasmids. *J Mol Biol* **166**, 557–580 (1983).
2. Bergès, H., Joseph-Liauzun, E. & Fayet, O. Combined effects of the signal sequence and the major chaperone proteins on the export of human cytokines in Escherichia coli. *Appl Environ Microbiol* **62**, 55–60 (1996).
3. Yasbin, R. E., Fields, P. I. & Andersen, B. J. Properties of Bacillus subtilis 168 derivatives freed of their natural prophages. *Gene* **12**, 155–159 (1980).
4. São-José, C., Baptista, C. & Santos, M. A. Bacillus subtilis operon encoding a membrane receptor for bacteriophage SPP1. *J Bacteriol* **186**, 8337–8346 (2004).
5. Okubo, S. & Yanagida, T. Isolation of a suppressor mutant in Bacillus subtilis. *J Bacteriol* **95**, 1187–1188 (1968).
6. Riva, S., Polsinelli, M. & Falaschi, A. A new phage of Bacillus subtilis with infectious DNA having separable strands. *J Mol Biol* **35**, 347–356 (1968).
7. Becker, B. *et al.* Head morphogenesis genes of the Bacillus subtilis Bacteriophage SPP1. *J Mol Biol* **268**, 822–839 (1997).
8. Seul, A. *et al.* Biogenesis of a Bacteriophage Long Non-Contractile Tail. *J Mol Biol* **433**, 167112–167134 (2021).
9. Behrens, B., Lüder, G., Behncke, M., Trautner, T. A. & Ganesan, A. T. The genome of B. subtilis phage SPP1. *Mol Gen Genet* **175**, 351–357 (1979).
10. Auzat, I., Dröge, A., Weise, F., Lurz, R. & Tavares, P. Origin and function of the two major tail proteins of bacteriophage SPP1. *Mol Microbiol* **70**, 557–569 (2008).
11. Dröge, A. & Tavares, P. In vitro Packaging of DNA of the Bacillus subtilis bacteriophage SPP1. *J Mol Biol* **296**, 103–115 (2000).
12. Auzat, I., Petitpas, I., Lurz, R., Weise, F. & Tavares, P. A touch of glue to complete bacteriophage assembly: the tail-to-head joining protein (THJP) family. *Mol Microbiol* **91**, 1164–1178 (2014).
13. Godinho, L. M. *et al.* The revisited genome of bacillus subtilis bacteriophage SPP1. *Viruses* **10**, (2018).
