# Supplementary material for: Dual function of a highly conserved bacteriophage tail completion protein essential for bacteriophage infectivity
Source: Commun Biol. 2024 May 16;7:590. doi: 10.1038/s42003-024-06221-6 (PMC11099176; doi:10.1038/s42003-024-06221-6)
Supplement: Supplementary file 3 — Description of Additional Supplementary Files [file 42003_2024_6221_MOESM3_ESM.pdf]

Description of Additional Supplementary Files

File name: Supplementary Data 1
Description: TCP homologs of SPP1, Lambda, T5, TP901-1, HK97, P2 and Mu bacteriophages

File name: Supplementary Data 2
Description: THJP homologs of SPP1, Lambda, T5, TP901-1, HK97, P2 and Mu bacteriophages

File name: Supplementary Data 3
Description: Source data for graphs.
