# Supplementary material for: Dual function of a highly conserved bacteriophage tail completion protein essential for bacteriophage infectivity
Source: Commun Biol. 2024 May 16;7:590. doi: 10.1038/s42003-024-06221-6 (PMC11099176; doi:10.1038/s42003-024-06221-6)
Supplement: Supplementary file 7 — Reporting Summary [file 42003_2024_6221_MOESM7_ESM.pdf]

nature portfolio

Corresponding author(s): Isabelle Auzat

Last updated by author(s): Apr 3, 2024

Reporting Summary

Nature Portfolio wishes to improve the reproducibility of the work that we publish. This form provides structure for consistency and transparency in reporting. For further information on Nature Portfolio policies, see our Editorial Policies and the Editorial Policy Checklist.

Statistics

For all statistical analyses, confirm that the following items are present in the figure legend, table legend, main text, or Methods section.

n/a	Confirmed	
☐	☒	The exact sample size (*n*) for each experimental group/condition, given as a discrete number and unit of measurement
☐	☒	A statement on whether measurements were taken from distinct samples or whether the same sample was measured repeatedly
☒	☐	The statistical test(s) used AND whether they are one- or two-sided<br>*Only common tests should be described solely by name; describe more complex techniques in the Methods section.*
☒	☐	A description of all covariates tested
☒	☐	A description of any assumptions or corrections, such as tests of normality and adjustment for multiple comparisons
☐	☒	A full description of the statistical parameters including central tendency (e.g. means) or other basic estimates (e.g. regression coefficient) AND variation (e.g. standard deviation) or associated estimates of uncertainty (e.g. confidence intervals)
☒	☐	For null hypothesis testing, the test statistic (e.g. *F*, *t*, *r*) with confidence intervals, effect sizes, degrees of freedom and *P* value noted<br>*Give P values as exact values whenever suitable.*
☒	☐	For Bayesian analysis, information on the choice of priors and Markov chain Monte Carlo settings
☒	☐	For hierarchical and complex designs, identification of the appropriate level for tests and full reporting of outcomes
☒	☐	Estimates of effect sizes (e.g. Cohen's *d*, Pearson's *r*), indicating how they were calculated

Our web collection on statistics for biologists contains articles on many of the points above.

Software and code

Policy information about availability of computer code

Data collection	-ÄKTA purifier with UNICORN 5.31 was used for protein purifications.<br>-StepOnePlus real-time PCR system (Applied Biosystems) was used for TSA.<br>-QuantStudio 12K Flex Real-Time PCR System (Life Technologies) was used for qPCR.<br>-T3-Thermocycler Biometra was used for DNA amplification.<br>-100 kV Tecnai 12 Spirit transmission electron microscope (Thermo Fisher, New York NY, USA) equipped with a K2 Base 4k x 4k camera (Gatan, Pleasanton CA, USA) was used for negative coloration.<br>-120 kV Tecnai 12 Spirit transmission electron microscope (FEI/Thermo Fisher Scientific, Waltham, MA, USA) equipped with a 4k x 4k F416 camera (TVIPS GmbH, Gauting, Germany) was used for adsorption to mica experiment.
Data analysis	Thermal Shift Assay analysis : StepOne software v2.2.<br>Primer-Blast tool from NCBI and the Primer Express 3.0 software (Life Technologies) were used to design the primers for qPCR.<br>Plots and statistical analysis were determined using Microsoft Excel or GraphPad Prism v10.2.2 for Supplementary Figure 3b.<br>Microsoft PowerPoint was used for figure preparation.

For manuscripts utilizing custom algorithms or software that are central to the research but not yet described in published literature, software must be made available to editors and reviewers. We strongly encourage code deposition in a community repository (e.g. GitHub). See the Nature Portfolio guidelines for submitting code & software for further information.

Data

Policy information about availability of data

All manuscripts must include a data availability statement. This statement should provide the following information, where applicable:

- Accession codes, unique identifiers, or web links for publicly available datasets
- A description of any restrictions on data availability
- For clinical datasets or third party data, please ensure that the statement adheres to our policy

GenBank X97918.2: Bacteriophage SPP1 complete nucleotide sequence.
All data supporting the findings of this study are included in the manuscript.

Human research participants

Policy information about studies involving human research participants and Sex and Gender in Research.

Reporting on sex and gender	NA
Population characteristics	NA
Recruitment	NA
Ethics oversight	NA

Note that full information on the approval of the study protocol must also be provided in the manuscript.

Field-specific reporting

Please select the one below that is the best fit for your research. If you are not sure, read the appropriate sections before making your selection.

☒ Life sciences ☐ Behavioural & social sciences ☐ Ecological, evolutionary & environmental sciences

For a reference copy of the document with all sections, see nature.com/documents/nr-reporting-summary-flat.pdf

Life sciences study design

All studies must disclose on these points even when the disclosure is negative.

Sample size	We did not use statistical methods to determine the sample sizes; they were chosen based on the standard in the field. The measurements were taken from distinct or independent biological samples.
Data exclusions	No data were excluded.
Replication	All experiments were repeated independently at least twice with similar results. The sample size (n) is provided in the figure legend and/or in the "Methods" section.
Randomization	No human or animals subjects were involved in this study so randomization is irrelevant.
Blinding	No human or animals subjects were involved in this study so blinding is irrelevant.

Reporting for specific materials, systems and methods

We require information from authors about some types of materials, experimental systems and methods used in many studies. Here, indicate whether each material, system or method listed is relevant to your study. If you are not sure if a list item applies to your research, read the appropriate section before selecting a response.

Materials & experimental systems

n/a	Involved in the study
☐	☒ Antibodies
☒	☐ Eukaryotic cell lines
☒	☐ Palaeontology and archaeology
☒	☐ Animals and other organisms
☒	☐ Clinical data
☒	☐ Dual use research of concern

Methods

n/a	Involved in the study
☒	☐ ChIP-seq
☒	☐ Flow cytometry
☒	☐ MRI-based neuroimaging

Antibodies

Antibodies used	-Polyclonal antibodies were produced by immunisation of rabbits with purified soluble (gp17 (Auzat et al. 2014) and gp19.1(Seul et al. 2023)) proteins at the INAF platform (Gif-sur-Yvette, France) and denatured (gp16.1 (Seul et al. 2023)) protein at FZB Biotechnik GmbH (Berlin, Germany).<br>-The secondary antibody (goat anti-rabbit, peroxidase-coupled, Sigma) was diluted 10,000-fold according to the manufacturer's instructions.<br>-Antigen/antibody complexes were detected by ECL or ECL prime western Blotting Reagents (GE Healthcare: RPN2106 or RPN2232) and medical X-ray films (Fuji: Super RX-N 47410 19289).
Validation	The specificity of each antibody was validated on bacterial extracts and on purified protein.
